# Synthesis of 6,7-Dihydro-1*H*,5*H*-pyrazolo[1,2-*a*]pyrazoles by Azomethine Imine-Alkyne Cycloadditions Using Immobilized Cu(II)-Catalysts

**DOI:** 10.3390/molecules26020400

**Published:** 2021-01-13

**Authors:** Urša Štanfel, Dejan Slapšak, Uroš Grošelj, Franc Požgan, Bogdan Štefane, Jurij Svete

**Affiliations:** Faculty of Chemistry and Chemical Technology, University of Ljubljana, Večna pot 113, 1000 Ljubljana, Slovenia; ursa.stanfel@gmail.com (U.Š.); dejan.slapsak@gmail.com (D.S.); Uros.Groselj@fkkt.uni-lj.si (U.G.); Franc.Pozgan@fkkt.uni-lj.si (F.P.); Bogdan.Stefane@fkkt.uni-lj.si (B.Š.)

**Keywords:** 1,3-dipolar cycloadditions, 2,3-dihydropyrazolo[1,2-*a*]pyrazoles, copper-catalyzed azomethine imine-alkyne cycloaddition (CuAIAC), azomethine imines, ynones

## Abstract

A series of 12 silica gel-bound enaminones and their Cu(II) complexes were prepared and tested for their suitability as heterogeneous catalysts in azomethine imine-alkyne cycloadditions (CuAIAC). Immobilized Cu(II)–enaminone complexes showed promising catalytic activity in the CuAIAC reaction, but these new catalysts suffered from poor reusability. This was not due to the decoordination of copper ions, as the use of enaminone ligands with additional complexation sites resulted in negligible improvement. On the other hand, reusability was improved by the use of 4-aminobenzoic acid linker, attached to 3-aminopropyl silica gel via an amide bond to the enaminone over the more hydrolytically stable *N*-arylenamine C-N bond. The study showed that silica gel-bound Cu(II)–enaminone complexes are readily available and suitable heterogeneous catalysts for the synthesis of 6,7-dihydro-1*H*,5*H*-pyrazolo[1,2-*a*]pyrazoles.

## 1. Introduction

**Figure 1:**
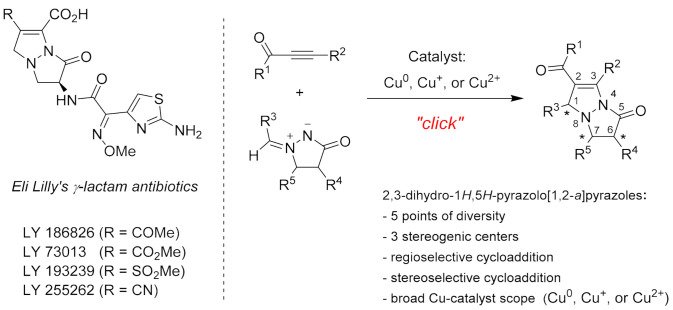
Examples of bioactive bicyclic pyrazolidinones (**left**) and CuAIAC reaction (**right**).

1,3-Dipolar cycloadditions of azomethine imines are important reactions to obtain pyrazoles with variable degree of saturation [1,2]. Since the end of the 20th century, this field has gained much attention; most azomethine imines have been recognized as stable compounds that are easy to prepare, store, and handle [1,2]. In this context, 1-alkylidene-3-oxopyrazolidin-1-ium-2-ides (3-oxopyrazolidin-1-azomethine imines), accessible by condensation of 1,2-unsubstituted pyrazolidin-3-ones with aldehydes or ketones, have been extensively used for regio- and stereoselective synthesis of pyrazolo[1,2-*a*]pyrazoles (bicyclic pyrazolidinones). Bicyclic pyrazolidinones exhibit antibiotic [3,4,5] and anti-Alzheimer activity [6], as well as inhibition of lymphocyte-specific protein tyrosine kinase [7,8] and *Plasmodium falciparum* dihydroorotate dehydrogenase (PfDHODH) [9]. The most prominent examples of bioactive bicyclic pyrazolidinones are Eli Lilly’s γ-lactam antibiotics, which exhibit antibiotic activity similar to that of penicillins and cephalosporins (Figure 1) [3,4,5]. These antibiotics are based on 6,7-dihydro-1*H*,5*H*-pyrazolo[1,2-*a*]pyrazole scaffold, which is accessible by [3 + 2] cycloaddition of 3-oxopyrazolidin-1-ium-2-ides to acetylenes [1,2]. In this context, copper-catalyzed azomethine imine-alkyne cycloadditions (CuAIAC) [1,2,10,11,12,13,14,15,16] provide easy access to 6,7-dihydro-1*H*,5*H*-pyrazolo[1,2-*a*]pyrazoles in a regio- and stereoselective manner under mild conditions that are compliant with requirements of “click” chemistry (Figure 1) [17,18,19,20,21,22,23]. In contrast to the CuAAC reaction, which is catalyzed only by Cu(I), the azomethine imine analogue (CuAIAC) is also catalyzed by Cu(II) [10,11,12,24,25,26,27]. This is a major advantage in terms of catalyst scope and simplicity of workup as the use of reducing agent, such as sodium ascorbate, can be avoided when Cu(II) catalyst is used (Figure 1).

Alkyl 2-substituted-3-(dimethylamino)propenoates and related enaminones are readily available and stable enamino-masked β-keto aldehydes, which are useful 1,3-dielectrophilic reagents in synthetic organic chemistry. Acid-catalyzed reactions with *N*-, *C*-, and *O*-nucleophiles take place under mild conditions by substitution of the dimethylamino group to give β-functionalized propenoates. With ambident nucleophiles, enaminones undergo cyclization into different heterocyclic systems [28,29,30,31,32,33]. Enaminones are also used as alkenes in cycloaddition reactions [34,35,36,37,38] and as bidentate N, O ligands [39,40,41,42,43,44,45,46,47,48,49] and tetradentate acacen-type ligands [27,50,51,52,53,54] to coordinate metal ions.

In recent years, an important part of our ongoing research on the chemistry of 3-pyrazolidinones [55] has been focused on CuAIAC reactions catalyzed by Cu(0) [56,57], Cu(I) [58,59,60,61], and Cu(II) [27]. In extension, we were interested in the use of immobilized Cu(II) complexes with enaminone-type ligands attached to the solid support in CuAIAC reactions. In contrast to the rather extensive use of immobilized copper complexes in azide-alkyne cycloadditions (CuAAC) [62], their applications in CuAIAC reactions are almost unknown [26]. 3-Aminopropyl silica gel-immobilized Cu(II)-enaminone complexes would be easy to prepare via a transamination reaction [28,29,30,31,32,33,63,64], could serve as heterogeneous Cu(II) catalysts for the synthesis of pyrazolo[1,2-*a*]pyrazoles, and would complement well the known examples of heterogeneous Cu(0)- [41], Cu(I)- [65,66,67,68,69], and Cu(II)-catalysts [26] in the CuAIAC reaction. Herein, we report the results of this study confirming the suitability of these new enaminone-based heterogeneous copper catalysts in regioselective [3 + 2] cycloadditions of 1-benzylidene-5,5-dimethyl-3-oxopyrazolidin-1-ium-2-ides to methyl propiolate leading to methyl 1-aryl-7,7-dimethyl-5-oxo-6,7-dihydro-1*H*,5*H*-pyrazolo[1,2-*a*]pyrazole-2-carboxylates.

## 2. Results

### 2.1. Synthesis and Catalytic Activity of Silica Gel-Bound Cu–Enaminone Complexes ***5a**–**g***

**Scheme 1:**
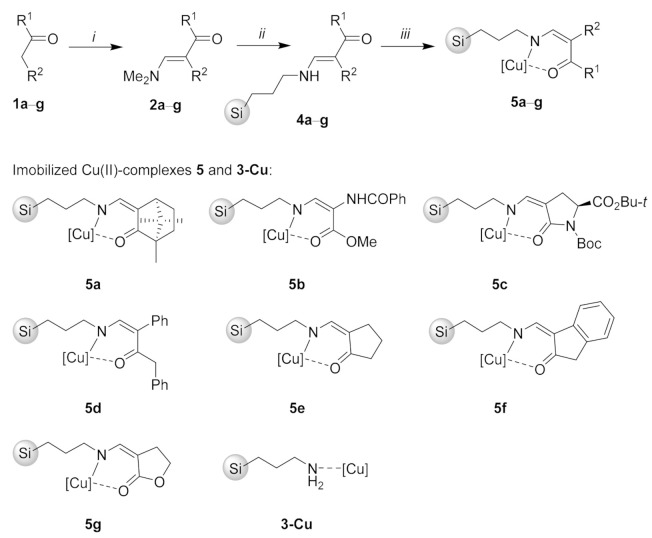
Reaction conditions: (i) DMFDMA or TBDMAM, CH_2_Cl_2_ or toluene, 20–110 °C; (ii) 3-aminopropyl silica gel (**3**), MeOH, 20 °C, 48 h; (iii) Cu(OAc)_2_·H_2_O, MeOH, 20 °C, 48 h.

First, the starting enaminones **2a**–**g** were prepared from active methylene compounds **1a**–**g** by treatment with *N*,*N*-dimethylformamide dimethylacetal (DMFDMA) or *tert*-butoxy-bis(dimethylamino)methane (TBDMAM) at 20–110 °C following literature procedure [27]. Next, the enaminones **2a**–**g** were reacted with equimolar amount of 3-aminopropyl silica gel (**3**) in methanol for 48 h to give the immobilized enaminones 4**a**–**g**. Subsequent treatment of 4**a**–**g** with one equivalent of Cu(OAc)_2_·H_2_O in methanol at room temperature for 48 h then furnished the desired complexes 5**a**–**g**. The complex **3**-**Cu** was prepared by treatment of **3** with Cu(OAc)_2_·H_2_O in methanol (Scheme 1). Absorption bands at around 1600 cm^−1^ (C = O/C = N) in the IR spectra of compounds **5a**–**h**, the results of combustion analyses for compounds 5**a**–**g**, and the results of characterization of the catalyst **5f** by SEM and EDX spectroscopy were in line with attachment of copper–enaminone complexes to **3** (For characterization details see the Supporting Information).

Compounds **5a**–**g** and **3**-**Cu** were then evaluated for their catalytic activity in [3 + 2] cycloaddition of (*Z*)-3,3-dimethyl-5-oxo-2-(3,4,5-trimethoxybenzylidene)pyrazolidin-2-ium-1-ide (**6a**) to methyl propiolate (**7**). The reaction was performed in CH_2_Cl_2_ at room temperature for 5 h with 30 mg (~20 mol%) catalyst loading (Table 1). Quantitative conversion was obtained only with 2-indanone-derived catalyst **5f** (Table 1, entry 6), while the conversion above 50% was also obtained from related enamino ketone-derived catalysts **5d** and **5e** (Table 1, entries 4 and 5). Catalysts 5**a**–**c** and **5g** were less active and the respective conversions ranged from 33% to 47% (Table 1, entries 1–3 and 7). Moderate activity of **3**-**Cu** (Table 1, entry 8) was in line with complexation of Cu(OAc)_2_ to 3-aminopropyl silica gel (**3**), which itself was found inactive (Table 1, entry 9).

The most active catalyst **5f** was tested further. The model reaction was carried out varying reaction time (1–3 h) and catalyst loading (10–30 mg). The results are presented in Table 2. In the presence of 30 mg of the catalyst, the conversion was around 50% after one hour, around 90% after two hours, and 100% after three hours (Table 2, entries 1–3). Complete conversion was also achieved with 25 mg and 20 mg of the catalyst (Table 2, entries 4 and 5), while further lowering of the catalyst loading to 15 mg (89%) and to 10 mg (61%) gave incomplete conversions (Table 2, entries 6 and 7).

**Scheme 2:**
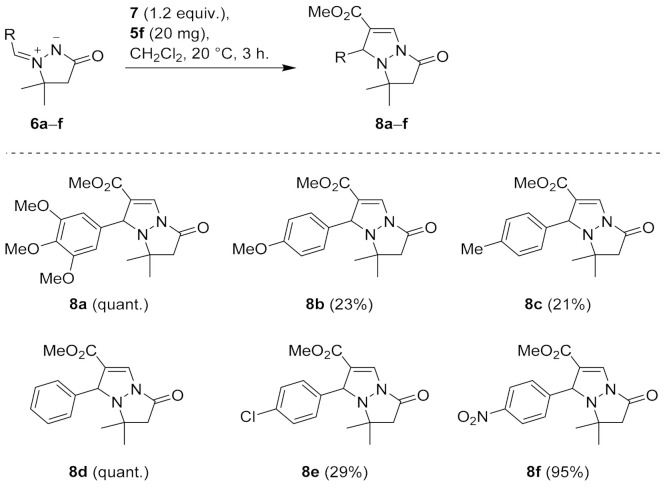
The conversions in CuAIAC reactions of dipoles **6a**–**f** with methyl propiolate (**7**) catalyzed by 20 mg (~13 mol%) of **5f**. The conversions were determined by ^1^H NMR of the crude reaction mixtures.

Next, the substrate scope was investigated using 20 mg (~13 mol%) of catalyst **5f** in reactions with azomethine imines **6a**–**f** (Scheme 2). After 3 h, only dipoles **6a** and **6d** were transformed quantitatively into the corresponding cycloadducts **8a** and **8d**, while conversions of other dipoles ranged from 23% to 95%. The highest conversions (95–100%) were obtained with 3,4,5-trimethoxyphenyl- (**6a**), phenyl- (**6d**), and 4-nitrophenyl-substituted dipole (**6f**), whereas poor conversions (23–29%) were observed with 4-methoxy- (**6b**), 4-methyl- (**6c**), and 4-chloro-substituted dipole (**6e**). Since closely related Cu^0^- and Cu^+^-catalyzed cycloadditions did not show any significant substrate dependence [56,57], incomplete conversions may seem surprising, yet they are explainable by much shorter reaction time (i.e., 12–48 h [56,57] vs. 3 h in the present case). Quantitative conversion of dipole **6e** into cycloadduct **8e** after 48 h was in line with this rationale (Scheme 2).

To further explore the reaction scope, azomethine imine **6a** was reacted also with nonpolar phenylacetylene in the presence of catalyst **5f** under the above standard reaction conditions. This reaction gave no conversion, even after prolonged treatment for 150 h. This result indicated a limitation of the reaction scope to polar electron-poor alkynes.

**Figure 2:**
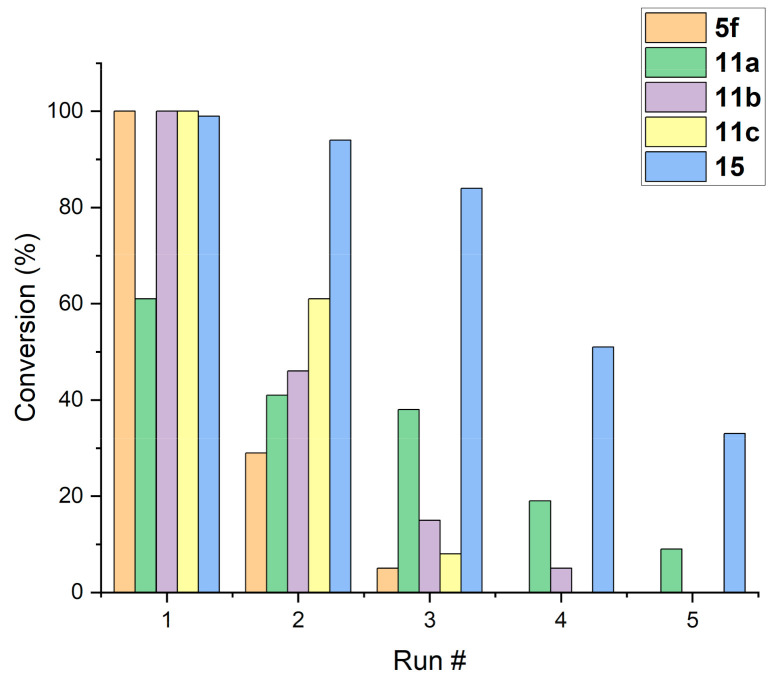
Reusability of catalysts **5f** (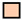), **11a** (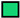), **11b** (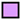), **11c** (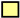), and **15** (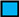) in the model reaction **6a** + **7** → **8a**. The conversions were determined by ^1^H NMR.

Reusability of the catalyst **5f** in the standard model reaction (**6a** + **7** → **8a**, 3 h, 30 mg of **5f**) was tested next. Much to our disappointment, the quantitative conversion in the first run dropped significantly in the second (29%) and the third run (5%) and the catalyst was inactive upon the third run (Figure 2). If poor reusability of catalyst **5f** is explainable by decomplexation of copper ions from the heterogeneous ligand **4f**, then reusability should be improved by stronger coordination of copper(II) to the ligand. Therefore, we decided to address the reusability issue by attaching stronger coordinating acacen ligands **9a**,**b** [27,50,51,52,53,54,70] and pyridine-enaminone ligands **9c** [71] to 3-aminopropyl silica gel (**3**).

### 2.2. Synthesis and Catalytic Activity of Silica Gel-Bound Cu–Enaminone Complexes ***11a**–**c*** and ***15***

**Scheme 3:**
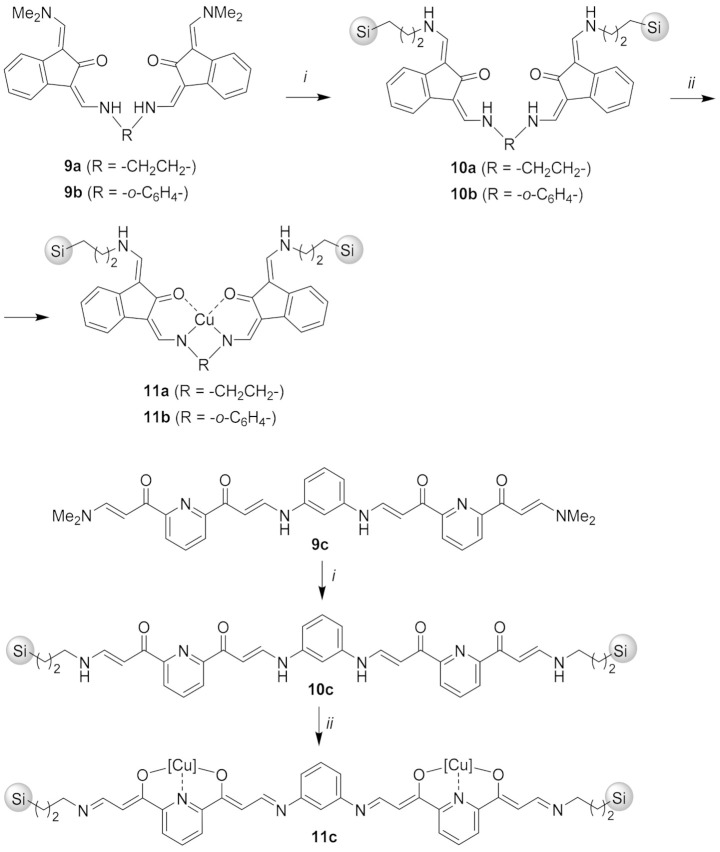
Reaction conditions: (i) 3-aminopropyl silica gel (**3**), MeOH, 20 °C, 48 h; (ii) Cu(OAc)_2_·H_2_O, MeOH, 20 °C, 48 h.

Bis-enaminone compounds **9a** and **9b** [70] (Scheme 3) contain two terminal *N,N*-(dimethyl)enaminone groups that enable transaminative attachment to 3-aminopropyl silica gel (**3**). Thus, treatment of **9a** and **9b** with **3** in methanol at room temperature afforded the immobilized acacen ligands **10a** and **10b**, which were subsequently reacted with Cu(OAc)_2_·H_2_O in methanol to furnish the desired immobilized Cu–acacen complexes **11a** and **11b** (Scheme 3). To obtain pyridine-type catalyst **11c**, bis-enaminone ligand **9c** [71], was reacted with 3-aminopropyl silica gel (**3**) to give silica gel-bound ligand **10c**, followed by treatment with Cu(OAc)_2_·H_2_O in methanol to furnish the copper complex **11c** (Scheme 3). Absorption bands at around 1600 cm^−1^ (C = O/C = N) in the IR spectra of compounds **11a**–**c** and the combustion analyses for compounds **11a**–**c** were in line with attachment of copper–enaminone complexes to **3** (For characterization details see the Supporting Information).

With the desired new catalysts **11a**–**c** in our hands, we first examined their catalytic activity in model cycloaddition (**6a** + **7** → **8a**, Table 3). After 3 h in the presence of 30 mg (~20 mol%) of the catalyst **11**, 1,2-ethylenediamine-based catalyst **11a** showed only moderate performance (61% conversion, Table 3, entry 1), while activities of 1,2-phenylenediamine-based catalyst **11b** and pyridine-based catalyst **11c** (Table 3, entries 2 and 3) were similar to that of catalyst **5f** (cf. Table 2, entry 3). Further evaluation of catalysts **11b** and **11c** in terms of catalyst loading (Table 3, entries 4–7) and reaction time (Table 3, entries 8–11) confirmed the performance of **11b** and **11c**, which was similar to that of catalyst **5f** (cf. Table 2, entries 1–7).

**Scheme 4:**
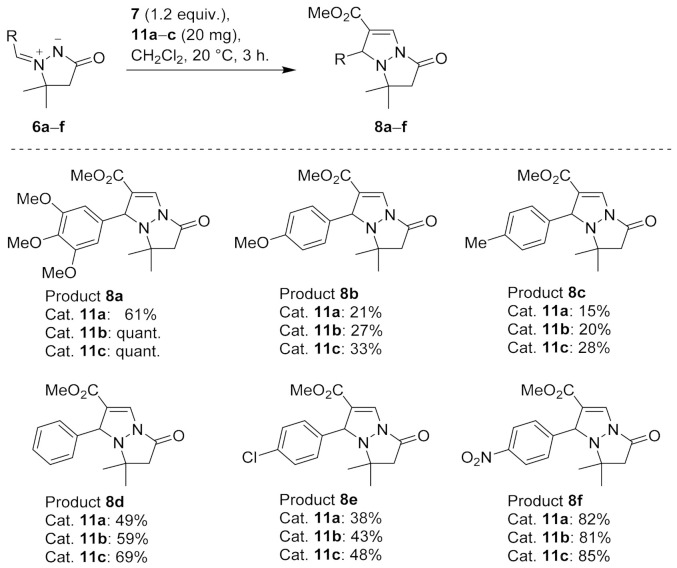
The conversions in CuAIAC reactions of dipoles **6a**–**f** with methyl propiolate (**7**) catalyzed by ~13 mol% of **11a–c**. The conversions were determined by ^1^H NMR of the crude reaction mixtures.

The substrate scope of catalysts **11a**–**c** was then checked by measuring conversions in the reactions of azomethine imines **6a**–**f** with methyl propiolate (**7**) in dichloromethane using ~13 mol% (20 mg) catalyst loading (Scheme 4). Quantitative conversions after 3 h were achieved only with dipole 6a in the presence of catalysts **11b** and **11c**, and with electron-poor dipole **6f**, relatively good conversions above 80% were obtained with all three catalysts. The conversions after 3 h were low to moderate (15–69%) with dipoles **6b**–**e**. For the most part, these results were in line with those obtained with catalyst **5f**. Notably, also the less reactive dipole 6e underwent full conversion within 48 h with catalyst **11c** (Scheme 4, cf. Scheme 3).

**Scheme 5:**
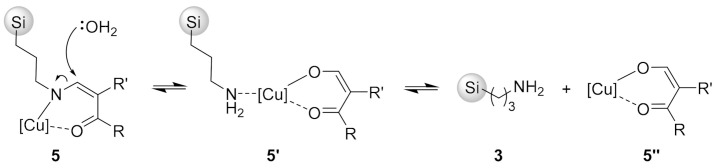
A plausible mechanism for detachment of Cu(II)-enaminone complex from **3**.

To our disappointment, reusability tests for catalysts **11a**–**c** in the standard model reaction (**6a** + **7** → **8a**, 3 h, 30 mg of **11**) revealed only minor improvement of reusability of catalysts **11a**–**c** in comparison to catalyst **5f**. Initially highly active catalysts **11b** and **11c** became inactive upon the third run (see Figure 2 at the end of Section 2.1). On the basis of these data, it became clear that decoordination of Cu(II) from the ligand was not the main reason for low reusability of **5f** and **11a**–**c**. We then considered that loss of catalytic activity could also be explainable by detachment of Cu(II)-enaminone complex from 3-aminopropyl silica gel (**3**), for example, through hydrolytic cleavage of the enamine C-N bond, as proposed in Scheme 5. Hydrolysis of enaminone complex **5** gives the complex **5′**, which can release Cu(II)-1,3-dicarbonyl complex **5″** in solution through decoordination from aminopropyl silica gel **3**.

**Scheme 6:**
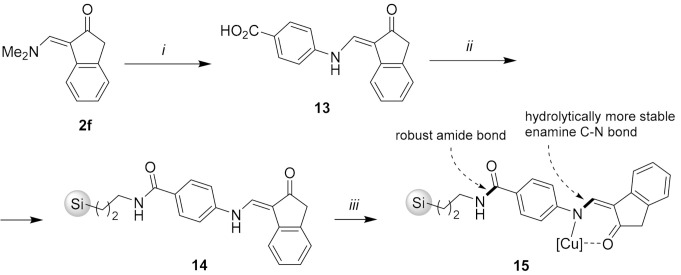
Reaction conditions: (*i*) 4-aminobenzoic acid (**12**), 37% aq. HCl (1 equiv.), MeOH, 20 °C; (*ii*) CDI, MeCN, 20 °C, 1 h, then 3-aminopropyl silica gel (**3**), MeCN, 20 °C, 120 h; (*iii*) Cu(OAc)_2_·H_2_O, MeOH, 20 °C, 48 h.

According to the proposed mechanism, the use of hydrolytically more stable enamine C-N bond should reduce detachment of Cu(II)-enaminone complex from the solid support and, thus, improve reusability of the catalyst. To confirm this hypothesis, we prepared silica gel-bound enaminone **14** using 4-aminobenzoic acid (**12**) as a bifunctional linker, which was bound to 3-aminopropyl silica gel (**3**) via a robust amide bond and to the enaminone **2f** through a stronger *N*-arylenamine C-N bond (Scheme 6) [28,29,30,31,32,33,63,64]. Acid-catalyzed transamination of **2f** with 4-aminobenzoic acid (**12**) gave the carboxy-functionalized enaminone **13**, which was amidated with **3** using 1,1′-carbonyldiimidazole (CDI) as activating reagent. Subsequent treatment of the silica gel-bound enaminone **14** with copper(II) acetate in methanol then furnished the desired catalyst **15** (Scheme 6). Absorption bands at around 1600 cm^−1^ (C = O/C = N) in the IR spectra of compound **15** and the combustion analyses for **15** were in line with attachment of copper–enaminone complex to **3** (For characterization details see the Supporting Information).

Activity and reusability of catalysts **5f**, **11a**–**c**, and **15** were tested in the standard model reaction (**6a** + **7** → **8a**, CH_2_Cl_2_, 20 °C, 3 h, 30 mg catalyst loading). The results are summarized in Figure 2. In the first run, quantitative conversion was obtained with catalysts **5f**, **11b**, **11c**, and **15**, while catalyst **11a** gave only 61% conversion. The catalytic activity of **5f** and **11b**,**c** dropped significantly and they became practically inactive after the second run. Surprisingly, the initially least active catalyst **11a** lost catalytic activity more slowly than analogues **5f** and **11b**,**c** and remained only weakly active in the fifth run. On the other hand, catalyst **15** gave a near quantitative conversion in the second run (94%), followed by a gradual decrease of catalytic activity leading to 31% conversion in the fifth run. Thus, the reusability of *N*-arylenaminone catalyst **15** was significantly better than that of *N*-alkylenaminone analogues **5** and **11** (Figure 2). This result was consistent with the hypothesis that the decrease of catalytic activity was largely due to the detachment of the copper-enaminone complex from the solid support by hydrolysis of the C-N bond of the enamine (cf. Scheme 5).

## 3. Conclusions

Transamination of enaminones **2a**–**g** and bis-enaminones **9a**–**c** with 3-aminopropyl silica gel (**3**) in methanol gives the corresponding silica gel-bound enaminones **4a**–**g** and **10a**–**c**. Subsequent treatment of the immobilized enaminones **4a**–**g** and **10a**–**c** with copper(II) acetate in methanol gives the corresponding silica gel-bound Cu(II) complexes **5a**–**g** and **11a**–**c**. Both reactions are general and take place with different types of enaminones **2** and **9** under mild conditions. The obtained copper(II) complexes **5a**–**g** and **11a**–**c** exhibit catalytic activity in azomethine imine-alkyne cycloadditions (CuAIAC). The 2-indanone-derived catalyst **5f** and the bis-enaminone-derived catalysts **11b** and **11c** showed the most promising activity, unfortunately, with poor reusability. The main cause of the poor reusability appears to be hydrolytic cleavage of the Cu(II)-enaminone complex from the 3-aminopropyl silica gel (**3**), rather than decomplexation of the copper(II) ions from the ligand. This hypothesis was confirmed by the synthesis of a modified catalyst **15** with hydrolytically more stable enamine C-N bond of the enamine attached to 3-aminopropyl silica gel (**3**) via a robust amide bond. Catalyst **15** exhibited better reusability while still retaining the same catalytic activity as analogues **5** and **11**. In conclusion, silica gel-bound Cu(II)-enaminone complexes **5**, **11**, and **15** are easily available heterogeneous catalysts for the regioselective synthesis of pyrazolo[1,2-*a*]pyrazoles via [3 + 2] cycloaddition of 3-pyrazolidinone-derived azomethine imines to terminal ynones.

## 4. Materials and Methods

### 4.1. General Information

All solvents and reagents were used as received. Melting points were determined on SRS OptiMelt MPA100—Automated Melting Point System (Stanford Research Systems, Sunnyvale, CA, USA). The ^1^H NMR, ^13^C NMR, and 2D NMR spectra were recorded in CDCl_3_ and DMSO-*d*_6_ as solvents using Me_4_Si as the internal standard on a Bruker Avance III UltraShield 500 plus instrument (Bruker, Billerica, MA, USA) at 500 MHz for ^1^H and at 126 MHz for ^13^C nucleus, respectively. IR spectra were recorded on a Bruker FTIR Alpha Platinum spectrophotometer (Bruker, Billerica, MA, USA). Microanalyses were performed by combustion analysis on a Perkin-Elmer CHN Analyzer 2400 II (PerkinElmer, Waltham, MA, USA). Mass spectra were recorded on **an** Agilent 6224 Accurate Mass TOF LC/MS (Agilent Technologies, Santa Clara, CA, USA). Parallel stirring was carried out on a Tehtnica Vibromix 313 EVT orbital shaker (400 rpm in all cases) (Domel, Železniki, Slovenia). Flash column chromatography was performed on silica gel (Silica gel 60, particle size: 0.035–0.070 mm, Sigma-Aldrich, St. Louis, MO, USA).

Active methylene compounds **1a**–**g**, 3-aminopropyl silica gel (**3**) (for preparative chromatography, 40–63 µm, 0.9 mmol/g amino groups, pore size ~9 nm), 4-aminobenzoic acid (**12**), Cu(OAc)_2_·H_2_O, *N,N*-dimethylformamide dimethylacetal (DMFDMA, for synthesis, ≥96%), *tert*-butoxy-bis(dimethylamino)methane (TBDMAM, technical grade), and 1,1′-carbonyldiimidazole (CDI) are commercially available (Sigma-Aldrich). Enaminones **2a** [72], **2b** [73], **2c** [74], **2d** [75], **2e** [76], **2f** [77], and **2g** [78], bis-enaminones **9a**, **9b** [70], and **9c** [71], and azomethine imines **6a**,**f** [79], **6b**,**e** [80], **6c** [81], and **6d** [82] were prepared following the literature procedures.

### 4.2. Synthesis of 3-Aminopropyl Silica Gel-Bound Copper(II)-Catalyst ***3**-**Cu***

A mixture of 3-aminopropyl silica gel (**3**) (5.015 g, 4.5 mmol of amino group), Cu(OAc)_2_·H_2_O (903 mg, 4.5 mmol), and methanol (25 mL) was stirred at 20 °C for 48 h. The insoluble material was collected by filtration, washed carefully with methanol until the filtrate was colorless (around 10 × 5 mL), and air-dried to give the copper(II) catalyst **3**-**Cu**. Blue powder (5.163 g).

### 4.3. General Procedure for the Synthesis of 3-Aminopropyl Silica Gel-Bound Copper(II) Catalysts ***5a**–**g***

Enaminone **2** (1.381 mmol) was added to a suspension of 3-aminopropyl silica gel (**3**) (1.534 g, 1.381 mmol of amino group) in methanol (4 mL) and the mixture was stirred at 20 °C for 48 h. The insoluble material was collected by filtration, washed with methanol until the filtrate was colorless (around 10 × 5 mL), and air-dried to give **4**. The immobilized enaminone **4** was resuspended in methanol (8 mL), Cu(OAc)_2_·H_2_O (275 mg, 1.381 mmol) was added, and the mixture was stirred at room temperature for 48 h. The insoluble material was collected by filtration, washed carefully with methanol until the filtrate was colorless (around 10 × 5 mL), and air-dried to give the copper(II) catalyst **5**. The following compounds were prepared in this manner:

#### 4.3.1. Compound **5a**

Prepared from **2a** (934 mg, 4.5 mmol) and **3** (5.015 g, 4.5 mmol of amino group) in MeOH (15 mL); then Cu(OAc)_2_·H_2_O (903 mg, 4.5 mmol), MeOH (25 mL). Blue powder (5.392 g), ν_max_ 1558 (C = O/C = N), 1418 cm^−1^.

#### 4.3.2. Compound **5b**

Prepared from **2b** (1.119 g, 4.5 mmol) and **3** (5.015 g, 4.5 mmol of amino group) in MeOH (15 mL); then Cu(OAc)_2_·H_2_O (903 mg, 4.5 mmol), MeOH (25 mL). Blue powder (5.611 g), ν_max_ 1558 (C = O/C = N), 1419 cm^−1^.

#### 4.3.3. Compound **5c**

Prepared from **2c** (1.119 g, 4.5 mmol) and **3** (5.015 g, 4.5 mmol of amino group) in MeOH (15 mL); then Cu(OAc)_2_·H_2_O (903 mg, 4.5 mmol), MeOH (25 mL). Blue powder (5.415 g), ν_max_ 1565 (C = O/C = N), 1418 cm^−1^.

#### 4.3.4. Compound **5d**

Prepared from **2d** (366 mg, 1.4 mmol) and 3 (1.534 g, 1.4 mmol of amino group) in MeOH (4 mL); then Cu(OAc)_2_·H_2_O (275 mg, 1.4 mmol), MeOH (8 mL). Blue powder (1.643 g), ν_max_ 1567 (C = O/C = N), 1416 cm^−1^.

#### 4.3.5. Compound **5e**

Prepared from **2e** (366 mg, 1.4 mmol) and 3 (1.534 g, 1.4 mmol of amino group) in MeOH (4 mL); then Cu(OAc)_2_·H_2_O (275 mg, 1.4 mmol), MeOH (8 mL). Light brown powder (1.598 g), ν_max_ 1565 (C = O/C = N), 1416 cm^−1^.

#### 4.3.6. Compound **5f**

Prepared from **2f** (844 mg, 4.5 mmol) and 3 (5.015 g, 4.5 mmol of amino group) in MeOH (15 mL); then Cu(OAc)_2_·H_2_O (903 mg, 4.5 mmol), MeOH (25 mL). Dark brown powder (5.514 g), ν_max_ 1606 (C = O/C = N), 1436 cm^−1^.

#### 4.3.7. Compound **5g**

Prepared from **2g** (195 mg, 1.4 mmol) and **3** (1.534 g, 1.4 mmol of amino group) in MeOH (4 mL); then Cu(OAc)_2_·H_2_O (275 mg, 1.4 mmol), MeOH (8 mL). Blue powder (1.607 g), ν_max_ 1565 (C = O/C = N), 1416 cm^−1^.

### 4.4. General Procedure for the Synthesis of Silica Gel-Bound Copper(II) Catalysts ***11a**–**c***

Bis-enaminone **9** (0.5 mmol) was added to a suspension of 3-aminopropyl silica gel (**3**) (1.111 g, 1 mmol of amino group) in methanol (4 mL) and the mixture was stirred at 20 °C for 48 h. The insoluble material was collected by filtration, washed with methanol until the filtrate was colorless (around 10 × 5 mL), and air-dried to give the silica gel-bound bis-enaminone **10**. The immobilized enaminone **10** was resuspended in methanol (8 mL), Cu(OAc)_2_·H_2_O (200 mg, 1 mmol) was added, and the mixture was stirred at room temperature for 48 h. The insoluble material was collected by filtration, washed carefully with methanol until the filtrate was colorless (around 10 × 5 mL), and air-dried to give the copper(II) catalyst **11**. The following compounds were prepared in this manner:

#### 4.4.1. Compound **11a**

Prepared from **9a** (100 mg, 0.2 mmol) and **3** (490 g, 0.4 mmol of amino group); then Cu(OAc)_2_·H_2_O (80 mg, 0.4 mmol). Brown powder (527 mg), ν_max_ 1569 (C = O/C = N), 1411 cm^−1^.

#### 4.4.2. Compound **11b**

Prepared from **9b** (200 mg, 0.4 mmol) and 3 (884 g, 0.8 mmol of amino group); then Cu(OAc)_2_·H_2_O (160 mg, 0.8 mmol). Green-blue powder (906 mg), ν_max_ 1564 (C = O/C = N), 1417 cm^−1^.

#### 4.4.3. Compound **11c**

Prepared from **9c** (322 mg, 0.56 mmol) and **3** (1.265 g, 1.12 mmol of amino group); then Cu(OAc)_2_·H_2_O (160 mg, 0.8 mmol). Brown powder (1.294 mg), ν_max_ 1552 (C = O/C = N), 1414 cm^−1^.

### 4.5. Synthesis of 3-Aminopropyl Silica Gel-Bound Copper(II) Complex ***15***

#### 4.5.1. (*E*)-4-{[(2-Oxo-2,3-dihydro-1*H*-inden-1-ylidene)methyl]amino}benzoic acid (**13**)

Enaminone **2f** (374 mg, 2 mmol) was added to a mixture of 4-aminobenzoic acid (**12**) (274 mg, 2 mmol), methanol (10 mL), and 37% aq. HCl (0.15 mL, 1.8 mmol) and the mixture was stirred at room temperature for 12 h. The precipitate was collected by filtration and washed with methanol (2 × 5 mL) and diethyl ether (2 × 5mL) to give **13**. Beige solid (385 mg, 69%); *E/Z* = 85:15; mp 263–264 °C (with slow decomposition above 200 °C); ν_max_/cm^−1^ (ATR) 3014, 2813, 1669 (C = O), 1594, 1566, 1424, 1261, 1180, 1199, 1092, 947, 848, 753, 713, 634; δ_H_ (500 MHz; DMSO-*d*_6_; Me_4_Si): major isomer 3.49 (2H, s), 7.09 (1H, td, *J* = 7.5, 1.1 Hz), 7.23–7.27 (2H, m), 7.52 (2H, d, *J* = 8.8 Hz), 7.67 (1H, d, *J* = 7.6 Hz), 7.92 (2H, d, *J* = 8.8 Hz), 8.39 (1H, d, *J* = 12.2 Hz), 11.03 (1H, d, *J* = 12.2 Hz), 12.74 (1H, s), minor isomer 3.46 (3H, s), 7.19 (1H, td, *J* = 7.5, 1.1 Hz), 7.23–7.27 (1H, m), 7.33 (2H, br d, *J* = 7.4 Hz), 7.48 (2H, br d, *J* = 8.8 Hz), 7.71 (1H, d, *J* = 13.4 Hz), 8.04 (1H, d, *J* = 7.4 Hz), 9.47 (1H, d, *J* = 13.3 Hz), 12.74 (1H, s); δ_C_ (126 MHz; DMSO-*d*_6_; Me_4_Si): major isomer 41.9, 111.5, 115.6, 117.6, 124.7, 124.8, 124.9, 126.9, 131.1, 134.3, 134.4, 140.0, 143.8, 166.8, 204.4, minor isomer 41.4, 112.8, 116.2, 121.9, 124.6, 124.7, 125.7, 126.8, 131.1, 131.8, 135.5, 138.2, 145.5, 166.9, 202.4; HRMS (ESI): MH^+^, found 280.0968 (MH^+^). [C_17_H_14_NO_3_]^+^ requires 280.0968; (found: C, 71.98; H, 4.24; N, 4.73. C_17_H_13_NO_3_·¼H_2_O requires C, 71.95; H, 4.79; N, 4.94%).

#### 4.5.2. Synthesis of Silica Gel-Bound Enaminone **14**

1,1′-Carbonyldiimidazole (85 mg, 0.52 mmol) was added to a stirred suspension of carboxylic acid **13** (140 mg, 0.5 mmol) in acetonitrile (5 mL) and the mixture was stirred at room temperature for 1 h. Then, 3-aminopropyl silica gel (**3**) (500 mg, 0.45 mmol of NH_2_ group) was added and the suspension was stirred at room temperature for 120 h. Ethanol (2 mL) was added and the insoluble material was collected by filtration using a short column with fritted bottom (d = 1.5 cm, l = 10 cm) and the functionalized silica gel **14** was washed with EtOH-MeCN (1:1, 3 × 5 mL), EtOH (2 × 5 mL), DMF (2 × 5 mL), EtOH (3 mL), and Et_2_O (2 × 5mL) and air-dried. Brown powder (536 mg, 31%, loading ~0.3 mmol/g); FT-IR (ATR): ν_max_ 1603 (C = O/C = N) cm^−1^.

#### 4.5.3. Synthesis of Silica Gel-Bound Copper(II) Catalyst **15**

3-Enaminopropyl silica gel **14** (300 mg, ~0.1 mmol of the enaminone) was added to a solution of Cu(OAc)_2_·H_2_O (50 mg, 0.25 mmol) in methanol (10 mL) and the mixture was stirred at 20 °C for 48 h. The insoluble material was collected by filtration, washed carefully with methanol until the filtrate was colorless (around 5 × 5 mL), and air-dried to give the copper(II) catalyst **15**. Brown powder (280 mg, 81%, loading ~0.3 mmol/g); FT-IR (ATR): ν_max_ 1603 (C = O/C = N) cm^−1^.

### 4.6. Synthesis of Methyl 1-Aryl-7,7-dimethyl-5-oxo-6,7-dihydro-1H,5H-pyrazolo[1,2-a]pyrazole-2-carboxylates ***8a**–**f*** by [3 + 2] Cycloadditions of Azomethine Imines ***6a**–**f*** to Methyl Propiolate (**7**) in the Presence of Catalysts ***3**-**Cu***, ***5***, ***11***, and ***15***

#### 4.6.1. Determination of Conversion. General Procedure A

Catalyst **3**-**Cu**, **5**, **11**, or **15** (10–30 mg) was added to a mixture of azomethine imine **6a**–**f** (25–37 mg [83], 0.125 mmol), methyl propiolate (**7**) (12.5 μL, 0.15 mmol), and CH_2_Cl_2_ (4 mL) and the mixture was stirred at room temperature for 1–5 h. The reaction mixture was filtered to remove the catalyst and the filtrate was evaporated in vacuo to give **8a**–**f** and ^1^H NMR spectrum of the residue was measured in CDCl_3_ to determine the conversion. ^1^H NMR data of compounds **8a**,**f** [79], **8d** [56,84], and **8e** [56] were in agreement with the literature data.

#### 4.6.2. Determination of Reusability of Catalysts. General Procedure B

Catalyst **5**, **11**, or **15** (30 mg) was added to a mixture of azomethine imine **6a** (37 mg, 0.125 mmol), methyl propiolate (**7**) (12.5 μL, 0.15 mmol), and CH_2_Cl_2_ (4 mL) and the mixture was stirred at room temperature for 3 h. Stirring was stopped, the catalyst was allowed to settle down for 2 min, and the supernatant was carefully decanted and filtered. Dichloromethane (4 mL) was added to the catalyst, the mixture was stirred for 2 min, the catalyst was allowed to settle down for 2 min, and the supernatant was carefully decanted and filtered. The catalyst was washed once more with dichloromethane (4 mL) as described above. The combined filtrate was evaporated in vacuo and ^1^H NMR spectrum of the residue was measured to determine conversion, while the washed catalyst was used in the next run.

#### 4.6.3. Synthesis of 1-aryl-7,7-dimethyl-5-oxo-6,7-dihydro-1*H*,5*H*-pyrazolo[1,2-*a*]pyrazole-2-carboxylates **8b** and **8c**. General Procedure C

Catalyst **5f** (120 mg) was added to a mixture of azomethine imine **6b** or **6c** (0.5 mmol), methyl propiolate (**7**) (50 μL, 0.6 mmol), and CH_2_Cl_2_ (5 mL) and the mixture was stirred at room temperature for 24 h. The catalyst was removed by filtration and washed with dichloromethane (3 mL). The combined filtrate was evaporated in vacuo and the residue was purified by flash column chromatography (Et_2_O). Fractions containing the product **8** were combined and evaporated in vacuo to give **8b** and **8c**.

*7,7-Dimethyl-1-(4-methoxyphenyl)-5-oxo-6,7-dihydro-1H,5H-pyrazolo[1,2-a]pyrazole-2-carboxylate* (**8b**). Prepared from azomethine imine **6b** (116 mg, 0.5 mmol), methyl propiolate (**7**) (50 μL, 0.6 mmol), and CH_2_Cl_2_ (4 mL). Yellow oil (74 mg, 47%); ν_max_/cm^−1^ (ATR) 2955, 1696 (C = O), 1599, 1511, 1444, 1408, 1371, 1323, 1200, 1173, 1099, 1031, 959, 824, 727; δ_H_ (500 MHz; CDCl_3_; Me_4_Si): 1.14 (3H, s), 1.22 (3H, s), 2.38 (1H, d, *J* = 15.7 Hz), 2.86 (1H, d, *J* = 15.7 Hz), 3.62 (3H, s), 3.79 (3H, s), 5.43 (1H, d, *J* = 1.3 Hz), 6.87 (2H, d, *J* = 8.7 Hz), 7.35 (2H, d, *J* = 8.7 Hz), 7.48 (1H, d, *J* = 1.3 Hz); δ_C_ (126 MHz; DMSO-*d*_6_; Me_4_Si): 19.1, 25.1, 49.6, 51.6, 55.3, 64.1, 64.4, 113.9, 117.1, 129.0, 129.3, 134.2, 159.3, 164.3, 166.5; HRMS (ESI): MH^+^, found 317.1493 (MH^+^). [C_17_H_21_N_2_O_4_]^+^ requires 317.1496.

*7,7-Dimethyl-1-(4-methylphenyl)-5-oxo-6,7-dihydro-1H,5H-pyrazolo[1,2-a]pyrazole-2-carboxylate* (**8c**). Prepared from azomethine imine **6c** (98 mg, 0.45 mmol), methyl propiolate (**7**) (50 μL, 0.6 mmol), and CH_2_Cl_2_ (4 mL). Yellow solid (83 mg, 61%); mp 152–155 °C; ν_max_/cm^−1^ (ATR) 3082, 2946, 1731 (C = O), 1687 (C = O), 1601, 1514, 1323, 1275, 1225, 1192, 1120, 1099, 1039, 1007, 947, 818, 733; δ_H_ (500 MHz; CDCl_3_; Me_4_Si): 1.12 (3H, s), 1.19 (3H, s), 2.33 (3H, s), 2.38 (1H, d, *J* = 14.7 Hz), 2.82 (1H, d, *J* = 14.7 Hz), 3.58 (3H, s), 5.40 (1H, d, *J* = 1.2 Hz), 7.12 (2H, d, *J* = 7.8 Hz), 7.29 (2H, d, *J* = 8.1 Hz), 7.46 (1H, d, *J* = 1.5 Hz); δ_C_ (126 MHz; DMSO-*d*_6_; Me_4_Si): 19.1, 21.3, 25.1, 49.5, 51.6, 64.4, 64.6, 117.0, 127.8, 129.2, 129.5, 137.6, 139.1, 164.3, 166.7; HRMS (ESI): MH^+^, found 301.1546 (MH^+^). [C_17_H_21_N_2_O_3_]^+^ requires 301.1547.

Following the above Procedure C, also known cycloadducts **8a**,**d**–**f** were obtained in the following isolated yields: compound **8a** (92%), **8d** (89%), **8e** (84%), and **8f** (88%). Spectral data for compounds **8a** [56,79], **8d** [56,84], **8e** [56], and **8f** [56,79] were in agreement with the literature data.

## Figures and Tables

**Table 1:** Evaluation of catalytic activity of 5**a**–**g**, **3**-**Cu**, and **3** in model cycloaddition reaction ^1^.

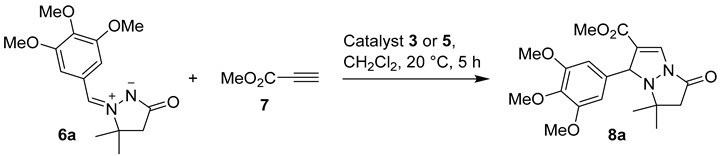					
**Entry**	**Catalyst**	**Conversion (%) ^2^**	**Entry**	**Catalyst**	**Conversion (%) ^2^**
1	**5a**	33	6	**5f**	quant.
2	**5b**	47	7	**5g**	39
3	**5c**	33	8	**3**-**Cu**	31
4	**5d**	64	9	**3**	0
5	**5e**	58			

^1^ Reaction conditions: **6a** (37 mg, 0.125 mmol), **7a** (13 mg, 0.150 mmol), catalyst **3** or **5** (30 mg, ~20 mol%), CH_2_Cl_2_ (4 mL), 20 °C, 5 h. ^2^ Determined by ^1^H NMR.

**Table 2:** Evaluation of catalyst **5f** in model cycloaddition reaction ^1^.

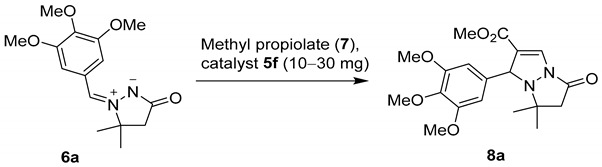			
**Entry**	**Loading (mg)**	**Time (h)**	**Conversion (%) ^2^**
1	30	1	51
2	30	2	89
3	30	3	quant.
4	25	3	quant.
5	20	3	97
6	15	3	89
7	10	3	61

^1^ Reaction conditions: **6a** (37 mg, 0.125 mmol), **7a** (13 mg, 0.150 mmol), catalyst **5f** (10–30 mg, ~7–20 mol%), CH_2_Cl_2_ (4 mL), 20 °C, 1–5 h. ^2^ Determined by ^1^H NMR.

**Table 3:** Catalytic activity of heterogeneous Cu(II) catalysts **11a**–**c** in model reaction ^1^.

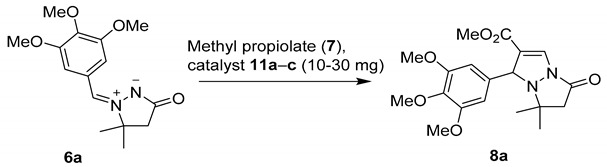				
**Entry**	**Catalyst 11**	**Loading (mg)**	**Time (h)**	**Conversion (%) ^2^**
1	**11a**	30	3	61
2	**11b**	30	3	quant.
3	**11c**	30	3	quant.
4	**11b**	20	3	quant.
5	**11c**	10	3	73
6	**11b**	20	3	quant.
7	**11c**	10	3	50
8	**11b**	30	2	quant.
9	**11b**	30	1	69
10	**11c**	30	2	quant.
11	**11c**	30	1	82

^1^ Reaction conditions: **6a** (37 mg, 0.125 mmol), **7** (13 mg, 0.150 mmol), catalysts **11a**–**c** (10–30 mg, ~7–20 mol%), CH_2_Cl_2_ (4 mL), 20 °C, 1–3 h. ^2^ Determined by ^1^H NMR.

## Data Availability

The data presented in this study are available in the Supporting Information.

## Supplementary Materials

The following are available online, copies of ^1^H and ^13^C NMR spectra of new compounds **8b**, **8c**, and **13**, copies of IR spectra of catalysts **5a**–**g**, **11a**–**c**, and **15**.

## ample Availability

Samples of the compounds **5a**–**g**, **8a**–**f**, **11a**–**c**, and **13**–**15** are available from the authors.
