# Supplementary material for: Synthesis of 6,7-Dihydro-1H,5H-pyrazolo[1,2-a]pyrazoles by Azomethine Imine-Alkyne Cycloadditions Using Immobilized Cu(II)-Catalysts
Source: Molecules. 2021 Jan 13;26(2):400. doi: 10.3390/molecules26020400 (PMC7828622; doi:10.3390/molecules26020400)
Supplement: Supplementary file 1 [file molecules-26-00400-s001.pdf]

molecules

MDPI

*Supporting Information for the manuscript:*

# Synthesis of 6,7-dihydro-1*H*,5*H*-pyrazolo[1,2-*a*]pyrazoles by azomethine imine–alkyne cycloadditions using immobilized Cu(II)-catalysts.

**Urša Štanfel, Dejan Slapšak, Uroš Grošelj, Franc Požgan, Bogdan Štefane, and Jurij Svete***

University of Ljubljana, Faculty of Chemistry and Chemical Technology, Večna pot 113, 1000 Ljubljana, Slovenia; jurij.svete@fkkt.uni-lj.si

* Correspondence: jurij.svete@fkkt.uni-lj.si; Tel.: +386-1-479-8562

Received: 9 December 2020; Accepted: 11 January 2021; Published: 13 January 2021

**Table of contents**

1. Copies of NMR spectra of compounds **8b**, **8c**, and **13**. 2–7

2. Copies of IR spectra of compounds **5**, **11**, and **15** 8–10

3. SEM images and EDX spectrum of compound **5f** 11–11

4. Elemental analyses for catalysts **5a–g** and **11a–c** 12–13

# 1. Copies of NMR spectra of compounds 8b, 8c, and 13.

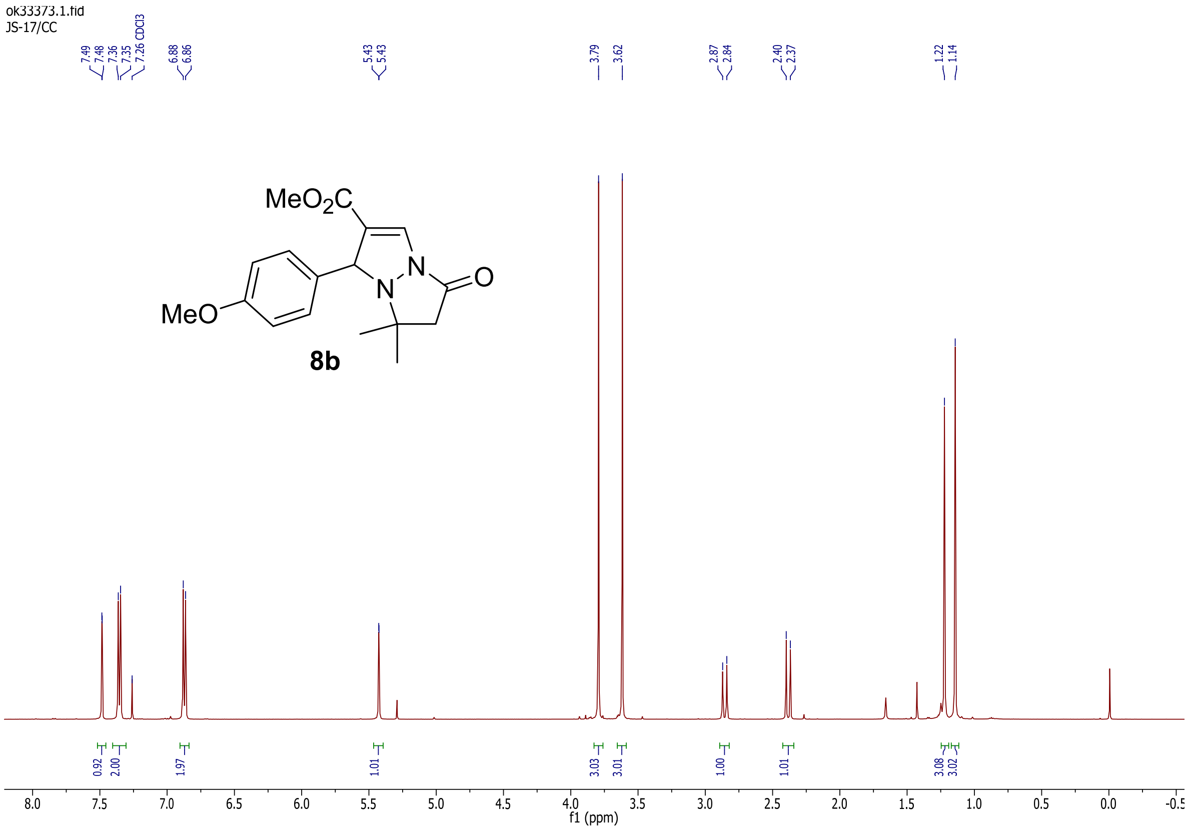


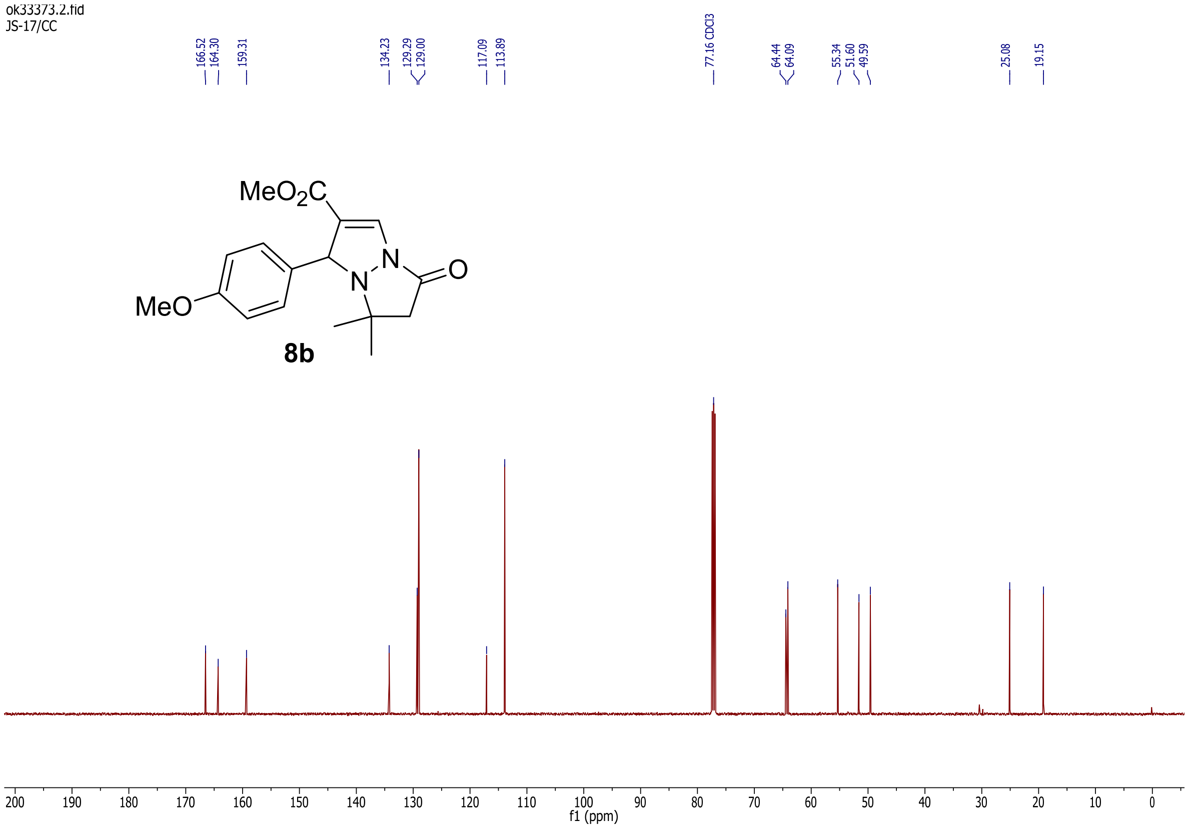

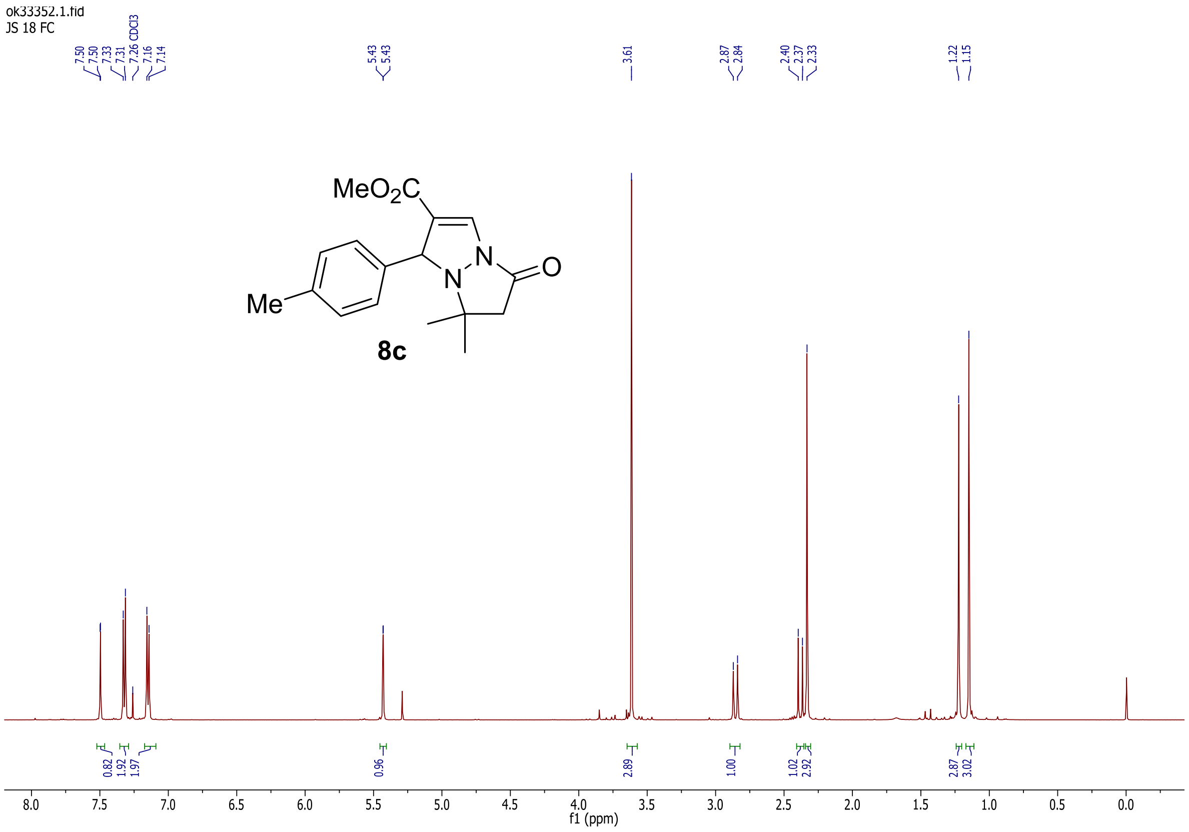
ok33352.1.fid
JS 18 FC
7.50 7.50 7.33 7.31 7.26 CDCl3 7.16 7.14
5.43 5.43
3.61
2.87 2.84
2.40 2.37 2.33
1.22 1.15
MeO2C
N
N
O
Me
8c
0.82 1.92 1.97
0.96
2.89
1.00
1.02 2.92
2.87 3.02
8.0 7.5 7.0 6.5 6.0 5.5 5.0 4.5 4.0 3.5 3.0 2.5 2.0 1.5 1.0 0.5 0.0
f1 (ppm)

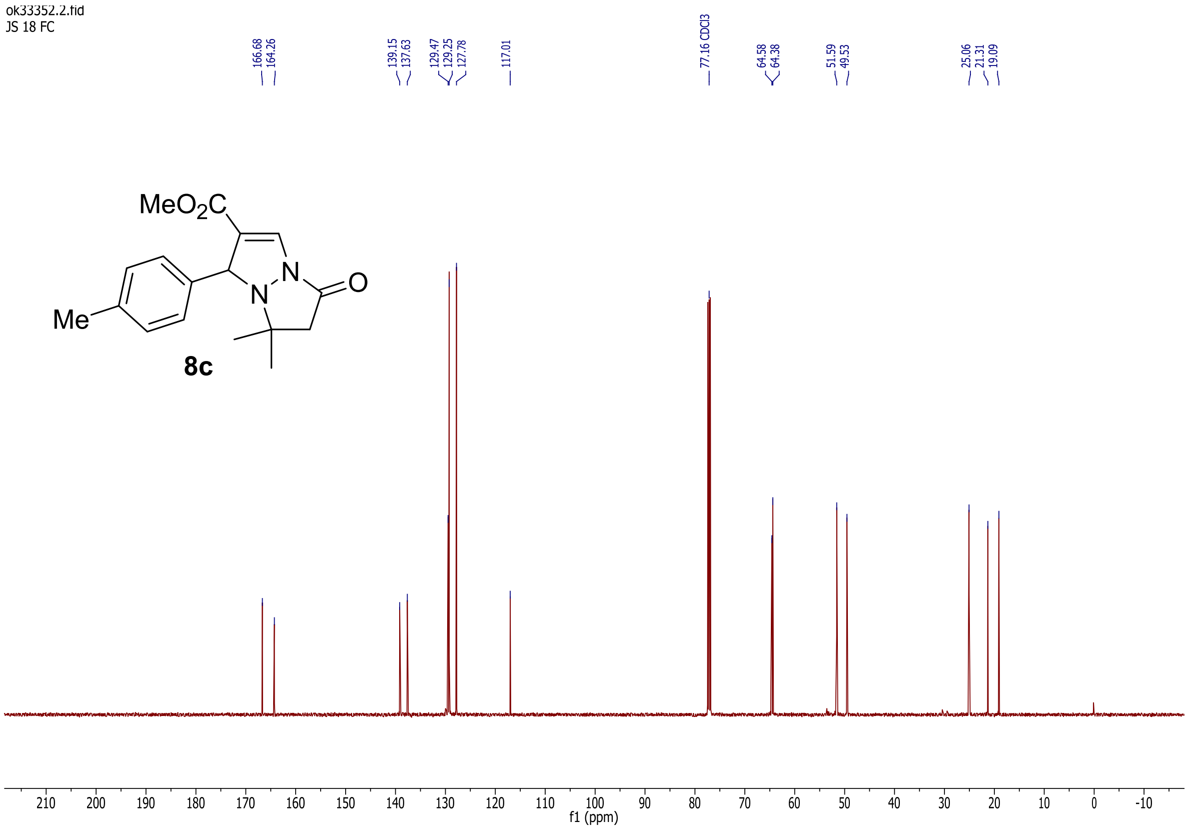
ok33352.2.fid
JS 18 FC
166.68 164.26
139.15 137.63 129.47 129.25 127.78
117.01
77.16 CDCl3
64.58 64.38
51.59 49.53
25.06 21.31 19.09
MeO2C
N
N
O
Me
8c
210 200 190 180 170 160 150 140 130 120 110 100 90 80 70 60 50 40 30 20 10 0 -10
f1 (ppm)

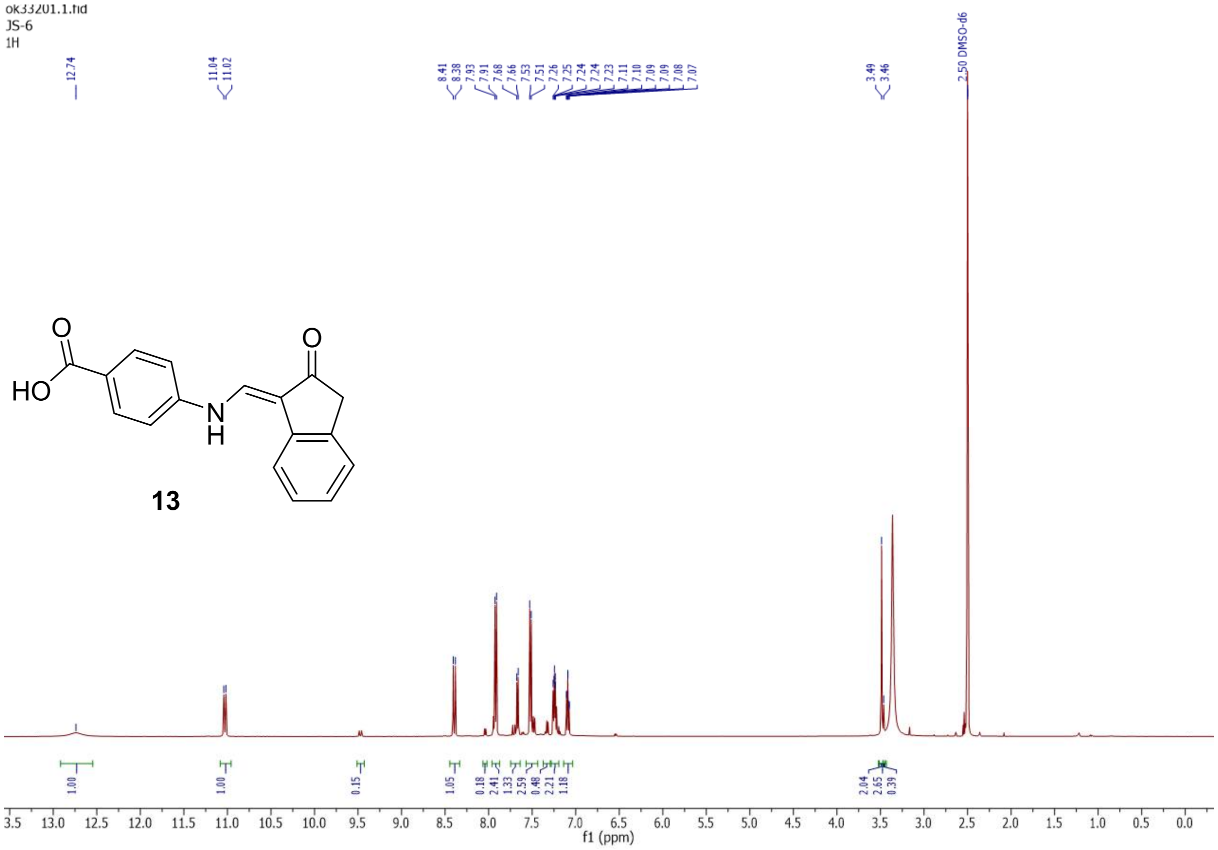
ok33201.1.fid
JS-6
1H
12.74
11.04
11.02
8.41
8.38
7.93
7.91
7.68
7.66
7.53
7.51
7.26
7.25
7.24
7.24
7.23
7.11
7.10
7.09
7.09
7.08
7.07
3.49
3.46
2.50 DMSO-d6
HO
O
N
H
O
13
1.00
1.00
0.15
1.05
0.18
2.41
1.33
2.59
0.48
2.21
1.18
2.04
2.65
0.39
f1 (ppm)

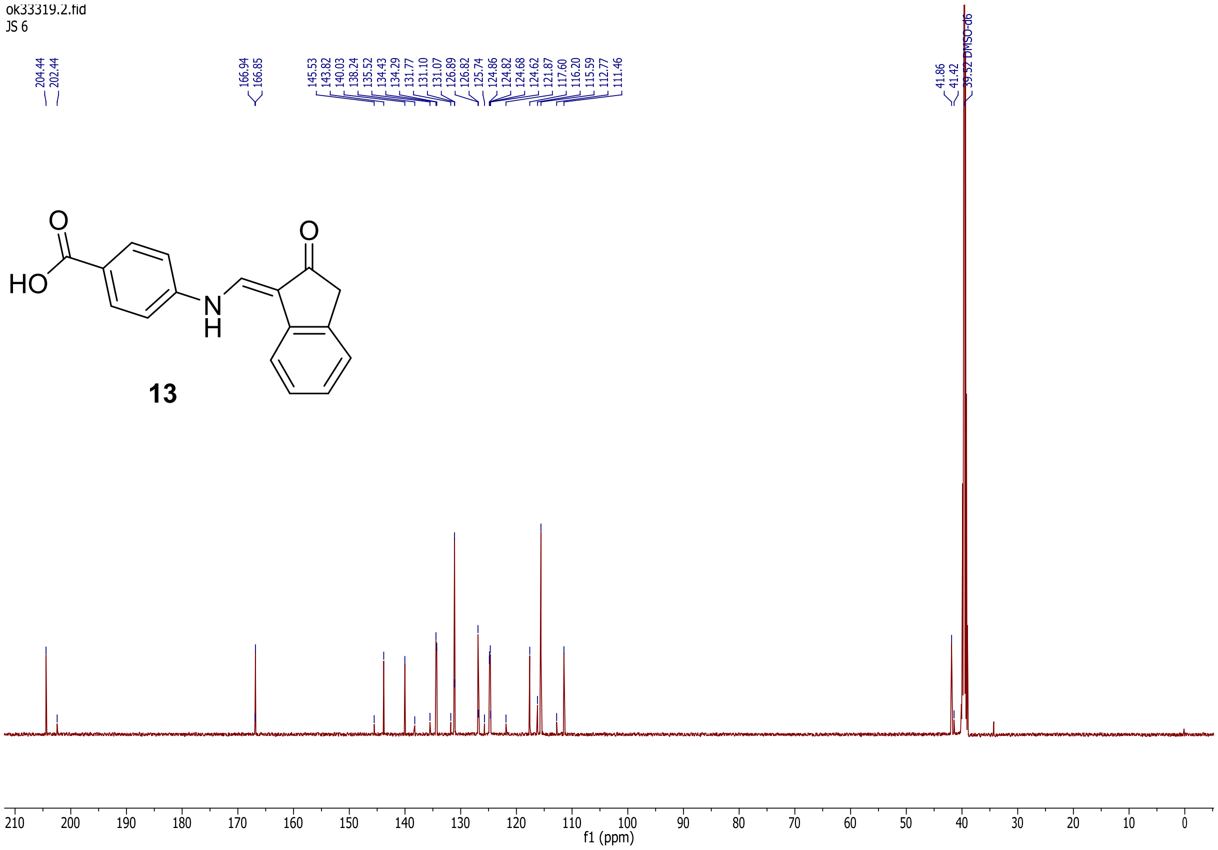
ok33319.2.fid
JS 6
204.44
202.44
166.94
166.85
145.53
143.82
140.03
138.24
135.52
134.43
134.29
131.77
131.10
131.07
126.89
126.82
125.74
124.86
124.82
124.68
124.62
121.87
117.60
116.20
115.59
112.77
111.46
41.86
41.42
39.52 DMSO-d6
HO
O
N
H
O
13
f1 (ppm)

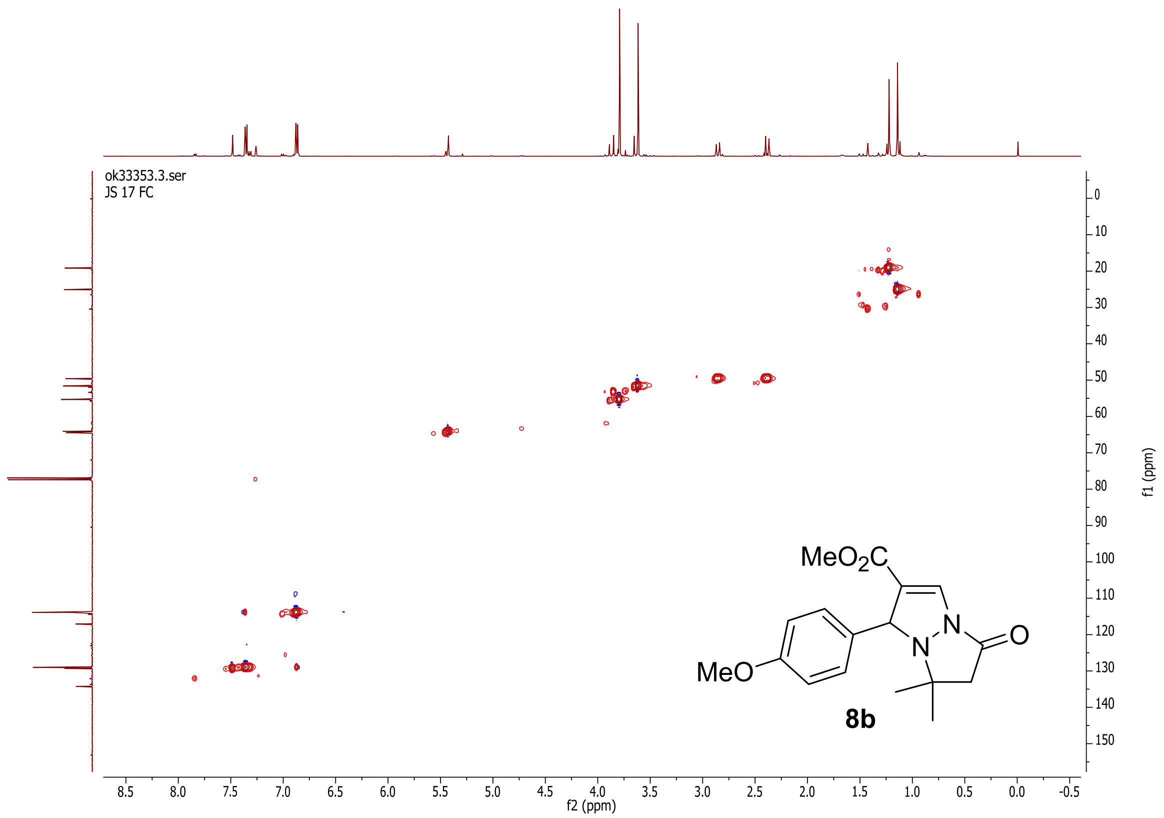
ok33353.3.ser
JS 17 FC
f2 (ppm)
f1 (ppm)
MeO2C
MeO
N
N
O
8b

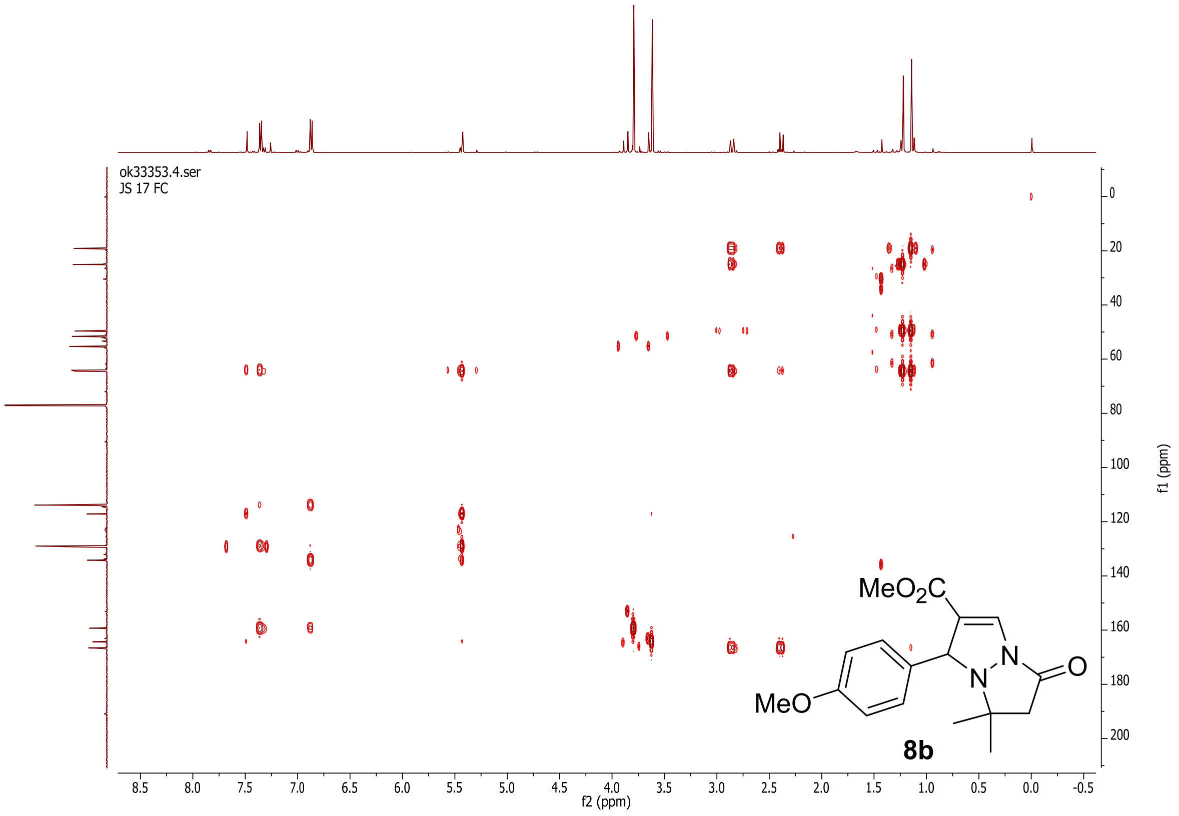
ok33353.4.ser
JS 17 FC
f2 (ppm)
f1 (ppm)
MeO2C
MeO
N
N
O
8b

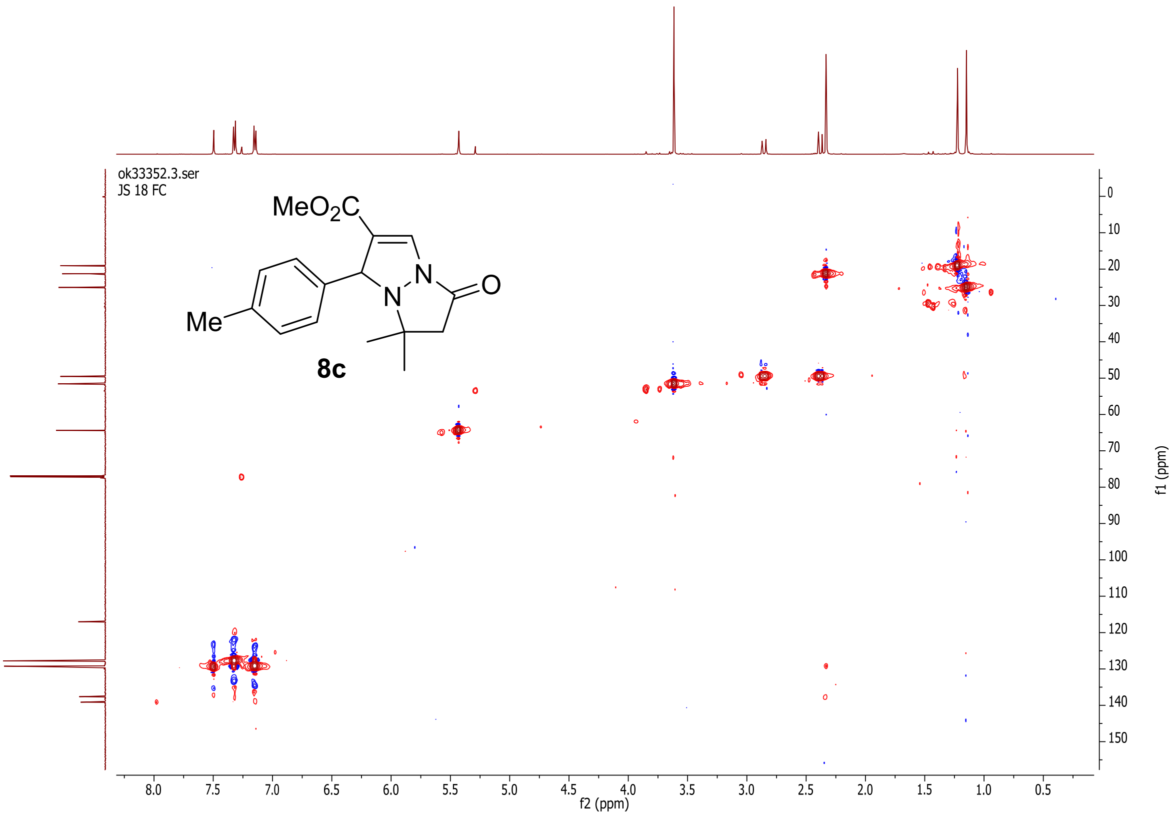
ok33352.3.ser
JS 18 FC
MeO2C
N
N
O
Me
8c
f2 (ppm)
f1 (ppm)

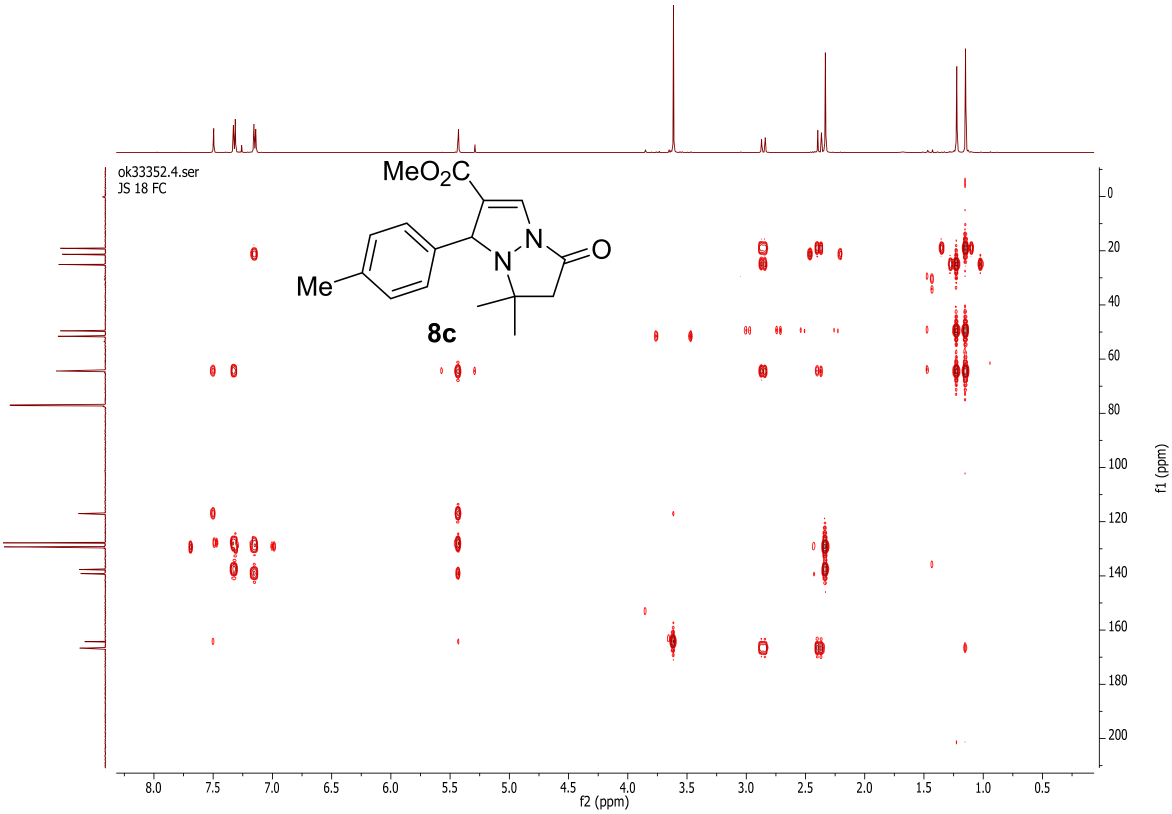
ok33352.4.ser
JS 18 FC
MeO2C
N
N
O
Me
8c
f2 (ppm)
f1 (ppm)

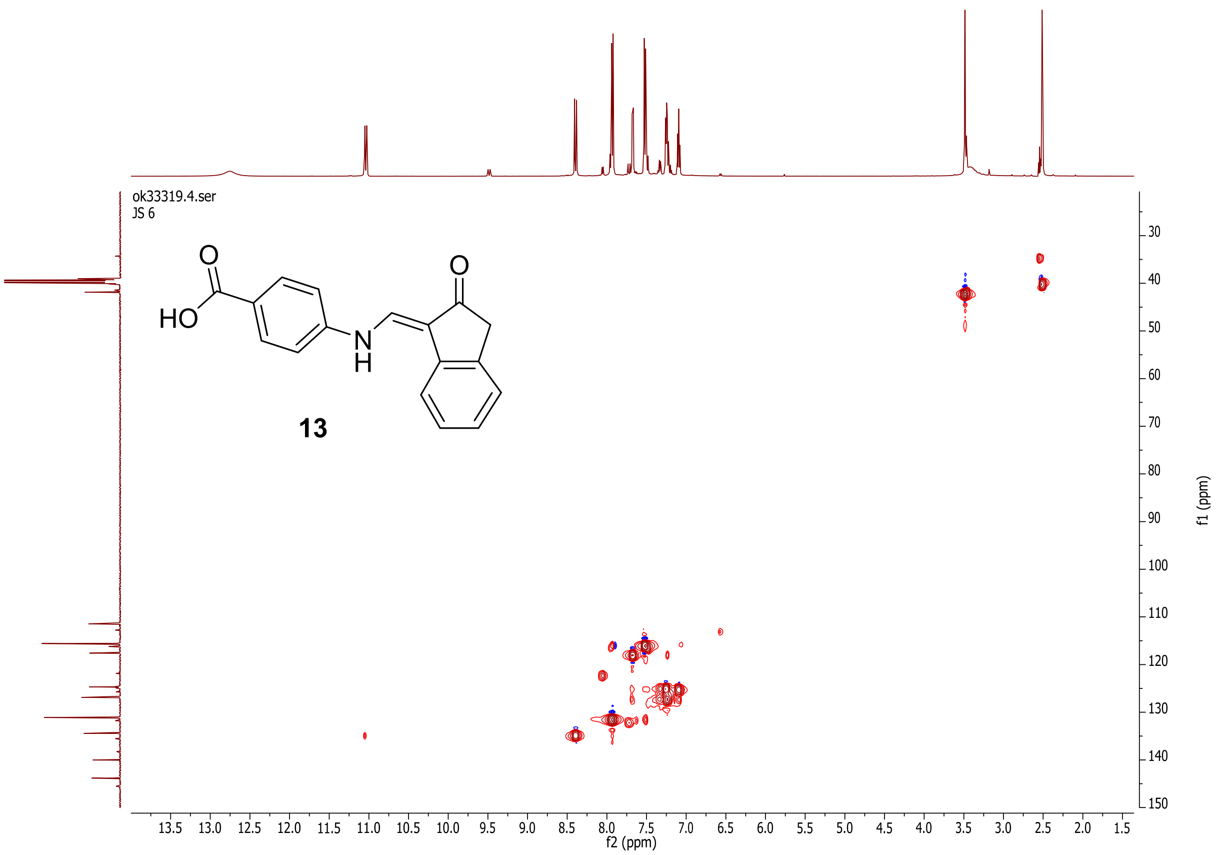
ok33319.4.ser
JS 6
HO
O
N
H
O
13
f2 (ppm)
f1 (ppm)

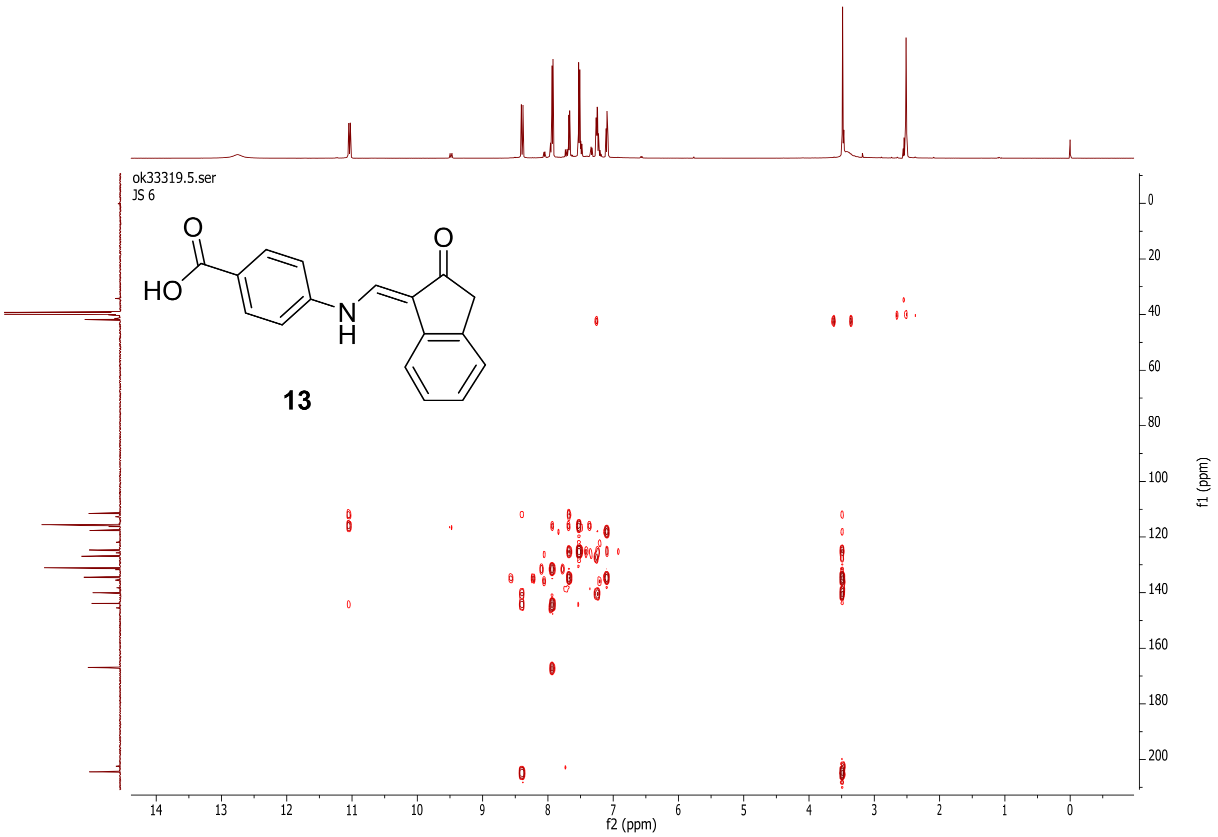
ok33319.5.ser
JS 6
HO
O
N
H
O
13
f2 (ppm)
f1 (ppm)

## 2. Copies of IR spectra of compounds 3, 5, 11, and 15.

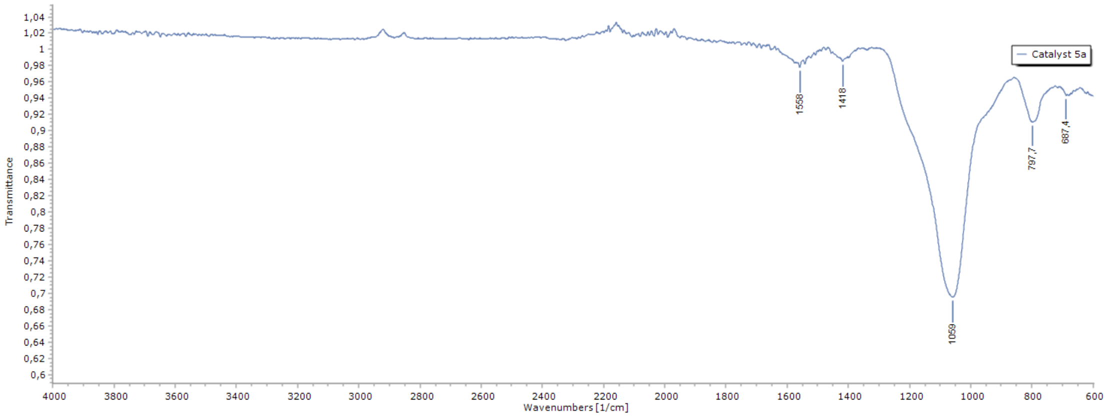


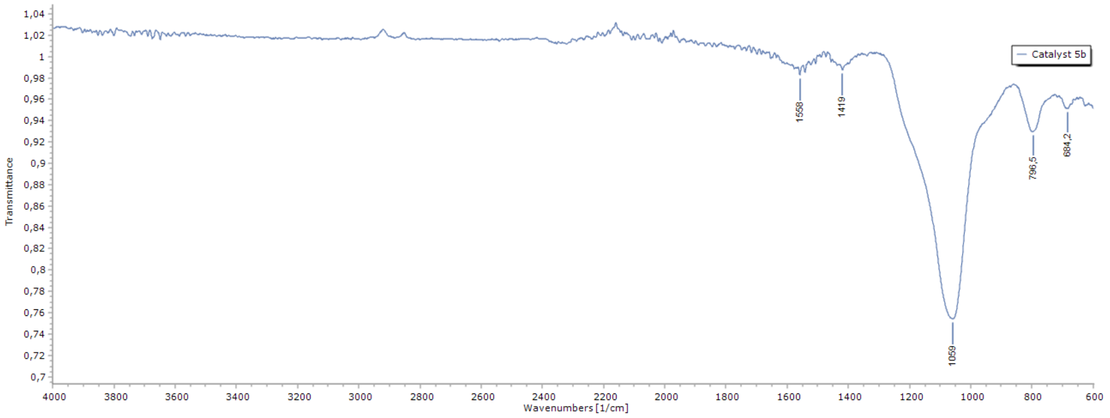


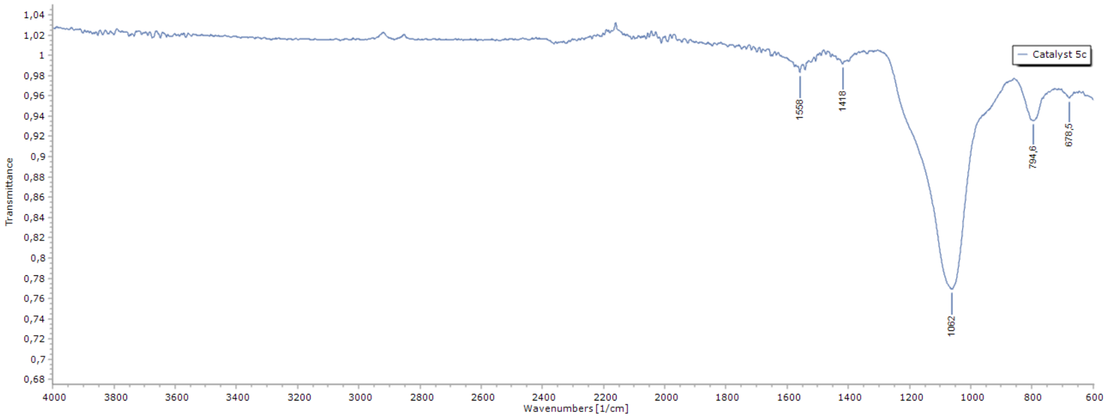


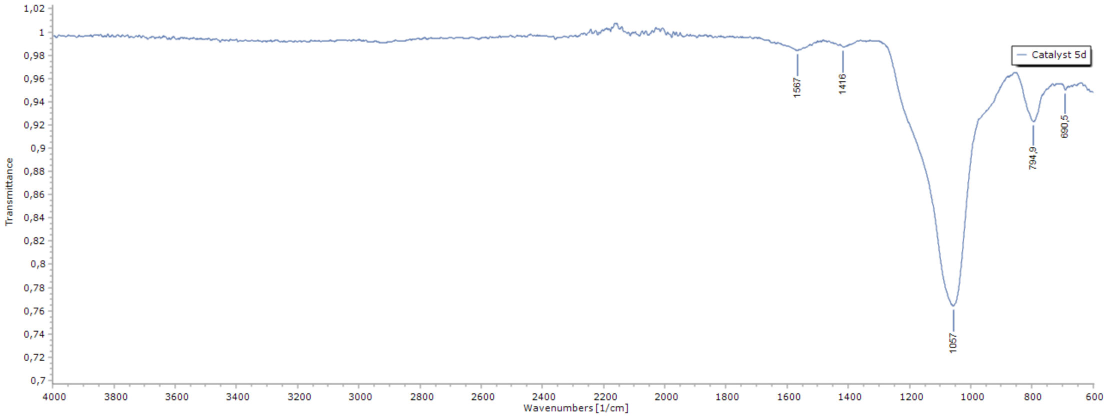

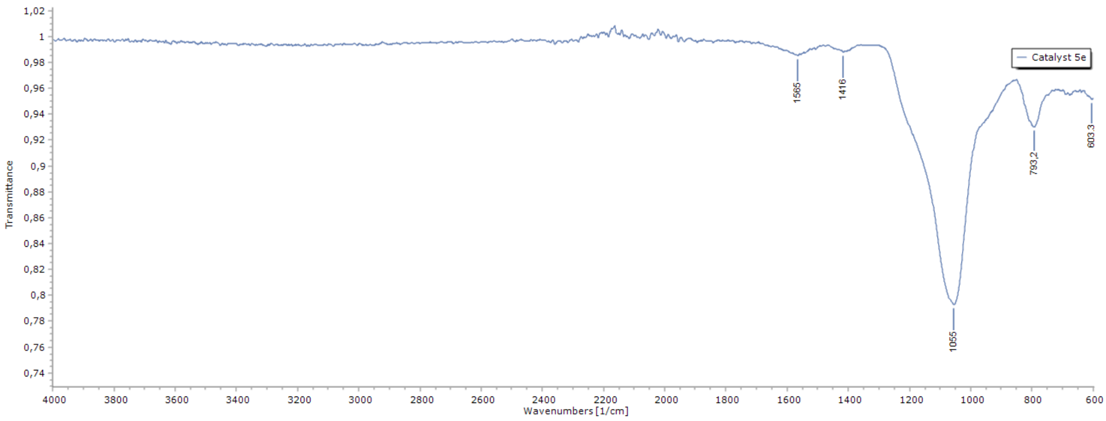
Catalyst 5e
1565
1416
1055
793,2
603,3
Transmittance
Wavenumbers [1/cm]

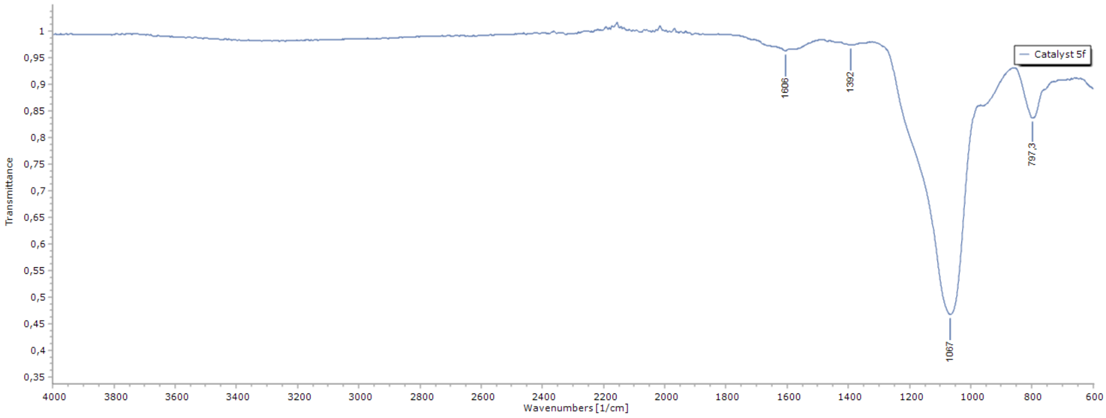
Catalyst 5f
1606
1392
1067
797,3
Transmittance
Wavenumbers [1/cm]

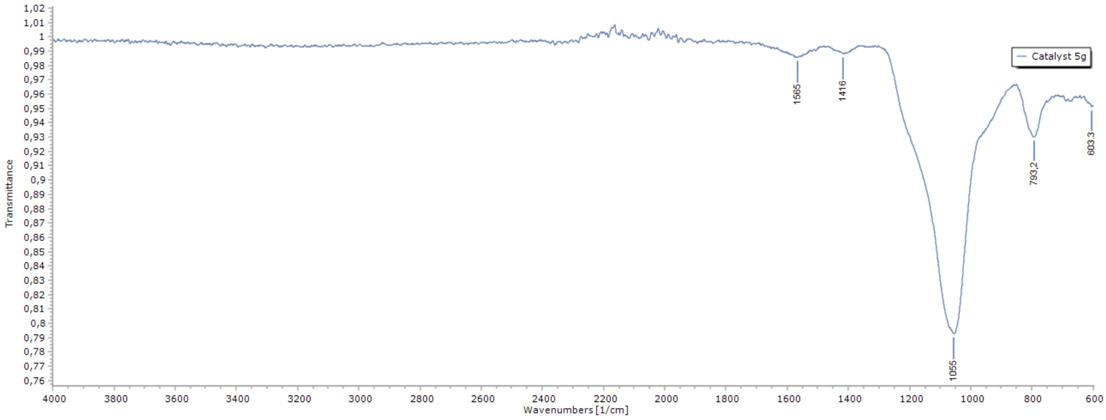
Catalyst 5g
1565
1416
1055
793,2
603,3
Transmittance
Wavenumbers [1/cm]

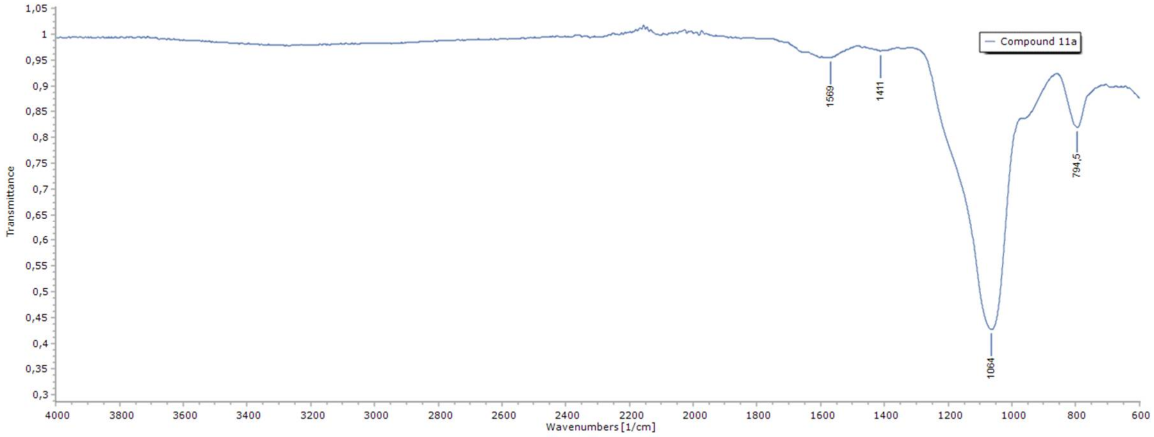
Compound 11a
1569
1411
1064
794,5
Transmittance
Wavenumbers [1/cm]

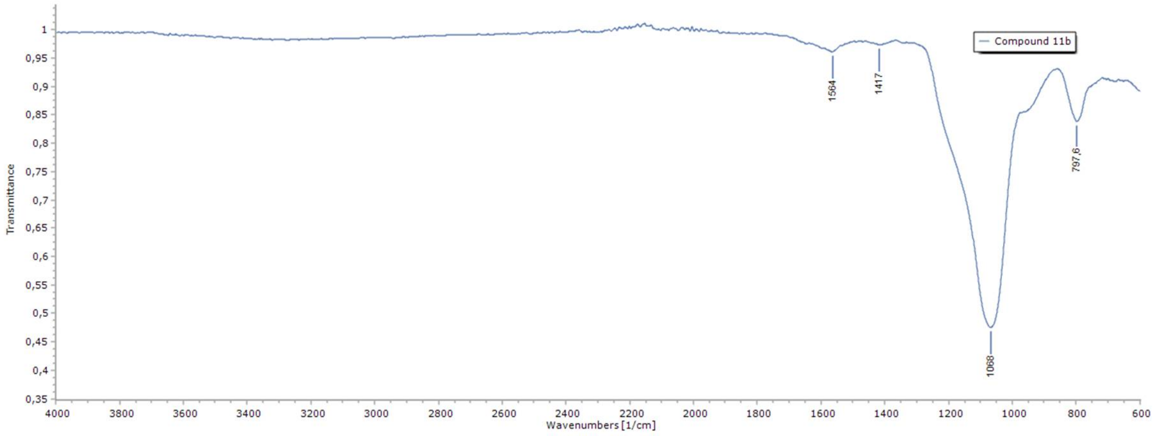
Compound 11b
Transmittance
Wavenumbers [1/cm]
1564
1417
1068
797,6

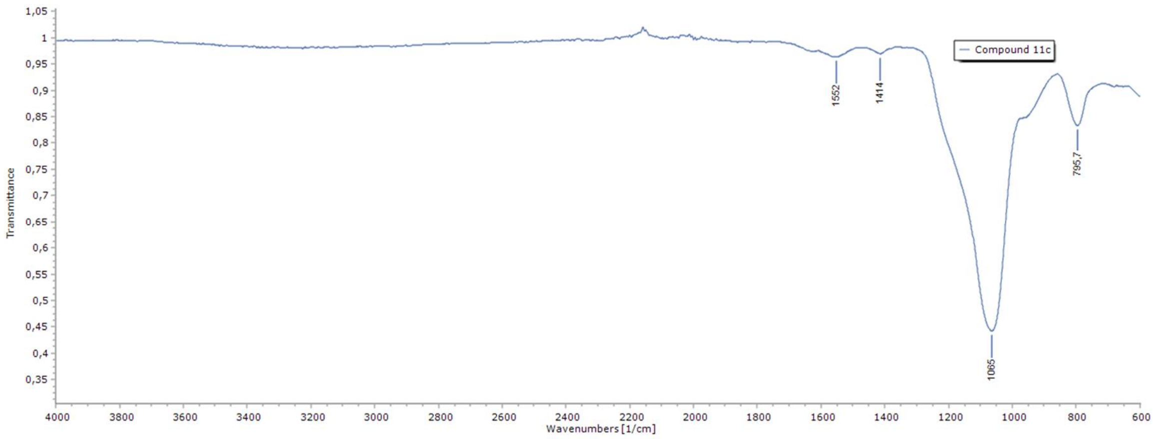
Compound 11c
Transmittance
Wavenumbers [1/cm]
1552
1414
1065
795,7

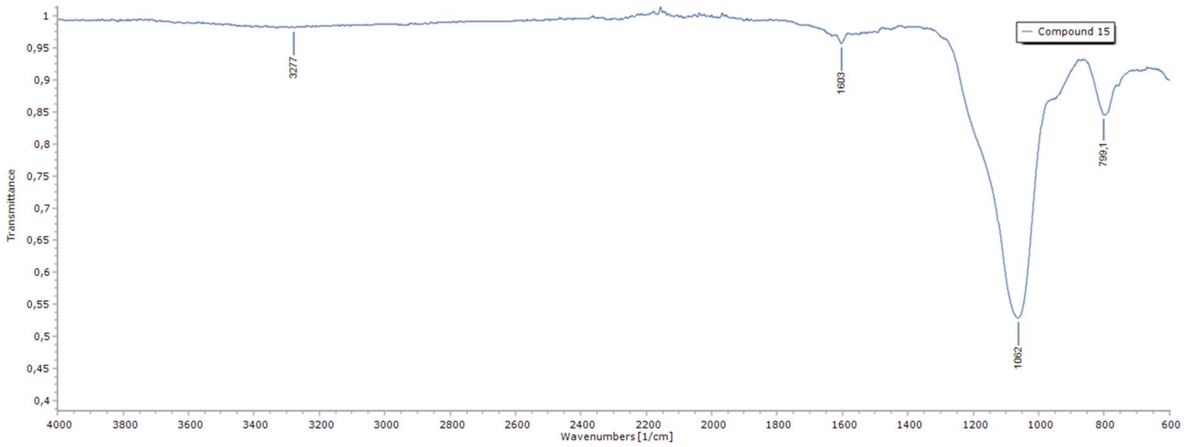
Compound 15
Transmittance
Wavenumbers [1/cm]
3277
1603
1062
799,1

## 3. SEM images and EDX spectrum of compound 5f.

SEM micrograph of **5f** (Figs. S1A) show narrow particle size distribution, which agrees with the homogeneity of particles in commercial 3-aminopropyl silica gel **3** (40–60 μm). The presence of copper in **5f** is in line with the observed signals in the EDX spectrum **5f** (Fig. S2).

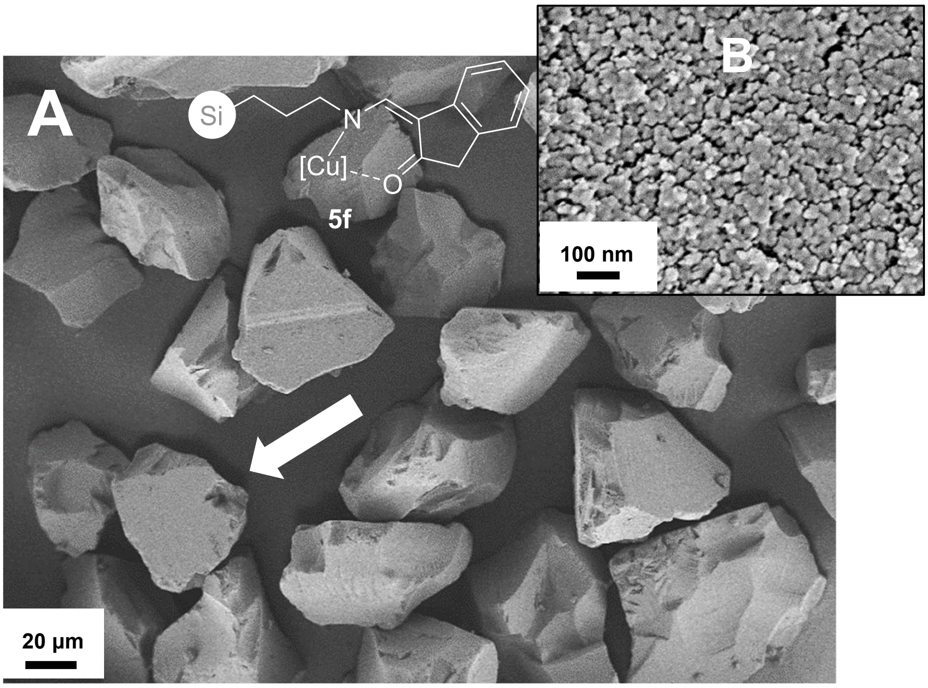


**Figure S1.** SEM micrographs showing morphology of catalyst **5f** at small magnification ($10^3$) - main micrograph (**A**) and large magnification ($2\times10^5$) – inset image **B** showing surface detail of a particle of **5f**.

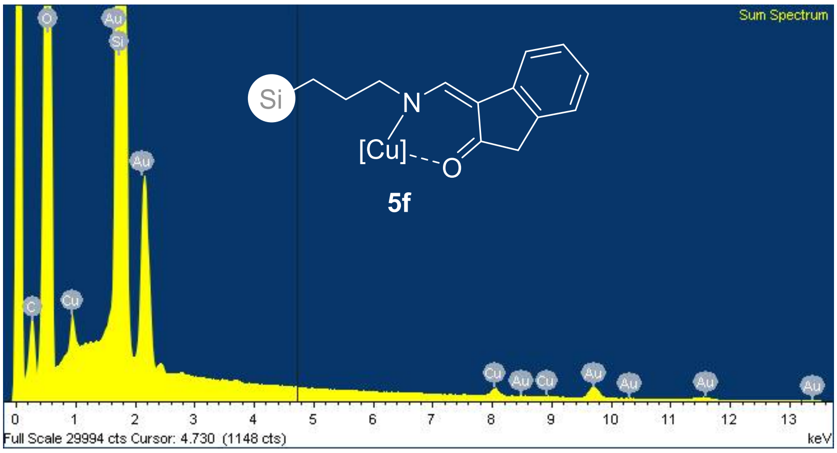


**Figure S2.** EDX spectrum taken from the surface of the above particle of **5f**.

## 4. Elemental analyses for catalysts 3-Cu, 5a–g, 11a–c, and 15.

To obtain more structural data on new catalysts, silica gel-bound copper complexes **3-Cu**, **5a–g**, **11a–c**, and **15** were characterized also by elemental analyses for C, H, and N. However, direct comparison of the found values for C, H, and N with the theoretical values for silica gel-bound copper complexes **3-Cu**, **5a–g**, **11a–c**, and **15** is not reasonable, due to variable degree of hydration of silica gel, which has a pronounced effect on the content of C, H, and N. On the other hand, silica gel itself does not contain any carbon or nitrogen, hence the C/N ratio of functionalized silica gels **3-Cu**, **5a–g**, **11a–c**, and **15** should depend only on the attached functional groups, such as aminopropyl residues in **3-Cu** and *N*-(propyl)enaminone residues in **5a–g**, **11a–c**, and **15**. We therefore decided to compare the found C/N ratios for **5a–g**, **11a–c**, and **15** with the calculated C/N ratios. The proposed structures **3-Cu**, **5a–g**, **11a–c**, and **15** used for characterization of silica gel-bound Cu(II) complexes are shown in Scheme S1, while the content of C, H, and N and the C/N ratios of **3-Cu**, **5a–g**, **11a–c**, and **15** are given in Table S1. The structure of **3-Cu** was proposed on the basis of the found C/N ratio of 3.91, which is in line with $Cu(OAc)_2$ coordinated with four propylamines (C/N = 3.43, Scheme S1, Table S1, entries 1–2). The enaminone–metal complexes **5a–g** and **15** should contain two bidentate enaminone ligands attached to Cu(II), while acacen complexes **11a** and **11b** should coordinate copper in 1:1 molar ratio. For catalyst **11c**, the structure with copper ion attached to the pyridine's nitrogen atom and two adjacent enol oxygens is proposed (Scheme S1).

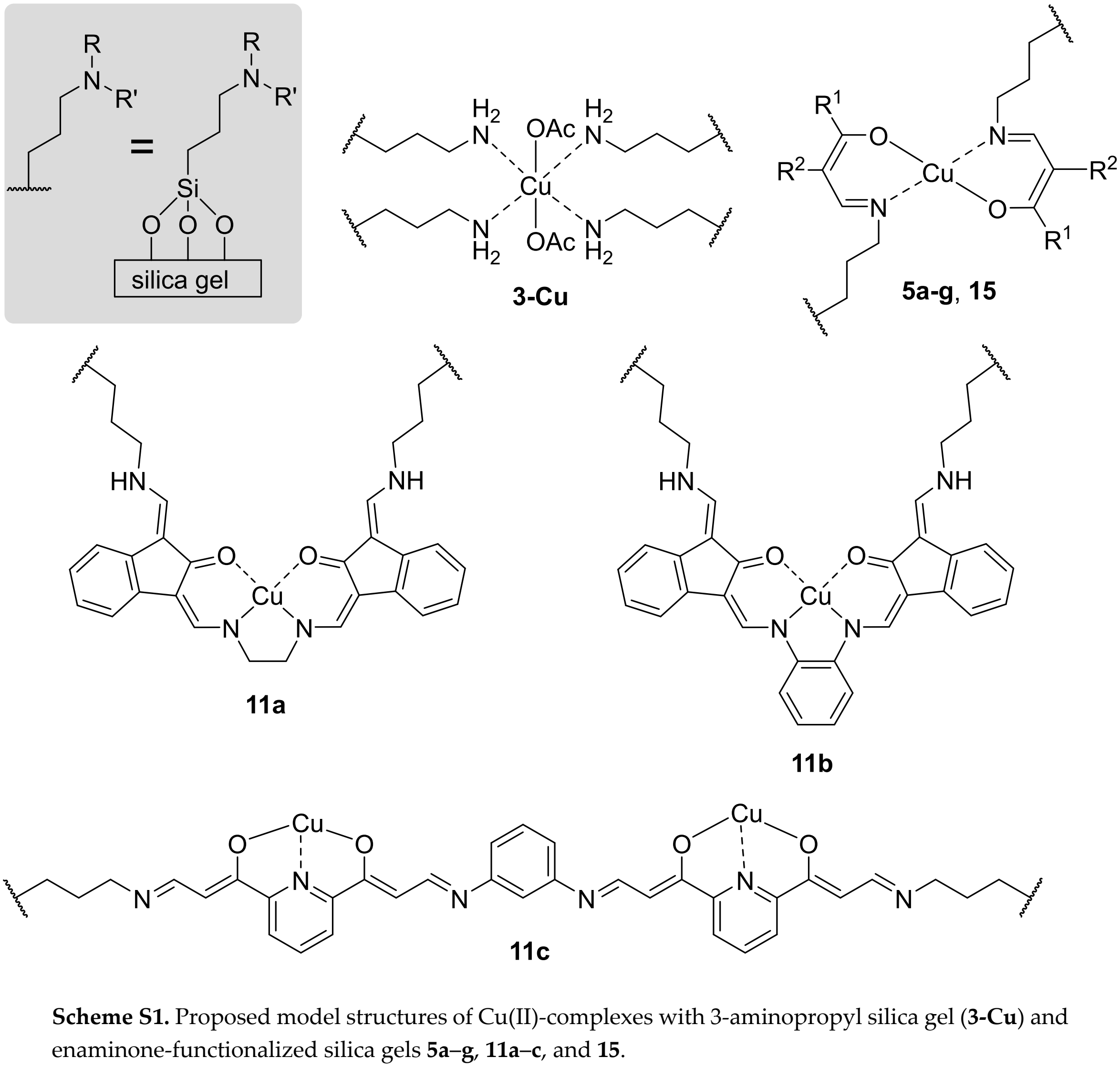


**Scheme S1.** Proposed model structures of Cu(II)-complexes with 3-aminopropyl silica gel (**3-Cu**) and enaminone-functionalized silica gels **5a–g**, **11a–c**, and **15**.

Elemental compositions and C/N ratios for catalysts **3-Cu**, **5a–g**, **11a–c**, and **15** are presented in Table S1. The calculated C/N ratio for **3-Cu** is 3.43 (entry 2), while for catalysts **5a–g**, **11a–c**, and **15** the calculated C/N ratios range between 4.86 for **11c** (entry 31) and 16.30 for **5d** (entry 13). The found C/N ratios for **11a** and **11c** (entries 24 and 30) are in good agreement with theoretical values (entries 25 and 31). Although the found values for catalysts **5a–g** (entries 3, 5, 9, 12, 15, 18, 21), **11b** (entry 26), and **15** (entry 32) did not match the theoretical values, they were all above 3.91, the found value for **3-Cu**. On the other hand, the found values for catalysts **5a–g**, **11b**, and **15** matched with the calculated values for mixtures of **3-Cu** with the respective complexes **5a–g** (entries 5, 8, 11, 14, 17, 20, 23), **11b** (entry 29), and **15** (entry 34). These results were in line with successful binding of copper–enaminones complexes to 3-aminopropyl silica gel (Table S1).

**Table S1.** Elemental analyses and C/N ratios for catalysts **3-Cu**, **5a–g**, **11a–c**, and **15**.

| Entry | Compound | Elemental composition (%) | | | C/N ratio |
|---|---|---|---|---|---|
| | | C | H | N | |
| 1 | **3-Cu (found)** | 5.24 | 1.16 | 1.34 | **3.91** |
| 2 | **3-Cu (calc.)** | 46.41 | 9.25 | 13.53 | **3.43** |
| 3 | **5a (found)** | 6.46 | 1.03 | 1.32 | **4.89** |
| 4 | **5a (calc.)** | 66.97 | 8.43 | 5.58 | 12.00 |
| 5 | **5a·2[3-Cu] (calc.)** | 54,17 | 8.94 | 10.53 | 5.14 |
| 6 | **5b (found)** | 6.55 | 1.27 | 1.33 | **4.92** |
| 7 | **5b (calc.)** | 57.57 | 5.52 | 9.59 | 6.00 |
| 8 | **5b 3-Cu (calc.)** | 52.94 | 7.07 | 11.23 | 4.71 |
| 9 | **5c (found)** | 9.61 | 1.07 | 1.71 | **5.62** |
| 10 | **5c (calc.)** | 56.27 | 7.35 | 7.29 | 7.72 |
| 11 | **5c·3-Cu (calc.)** | 52.82 | 8.01 | 9.84 | 5.37 |
| 12 | **5d (exp.)** | 7.71 | 1.49 | 1.21 | **6.37** |
| 13 | **5d (calc.)** | 73.82 | 6.2 | 4.53 | 16.30 |
| 14 | **5d·2[3-Cu] (calc.)** | 58.13 | 7.94 | 9.68 | 6.01 |
| 15 | **5e (found)** | 8.13 | 1.63 | 1.86 | **4.37** |
| 16 | **5e (calc.)** | 59.08 | 7.16 | 7.65 | 7.72 |
| 17 | **5e·2[3-Cu] (calc.)** | 50.29 | 8.61 | 1 | 4.29 |
| 18 | **5f (found)** | 11.88 | 1.35 | 1.26 | **9.43** |
| 19 | **5f (calc.)** | 67.59 | 5.67 | 6.06 | 11.15 |
| 20 | **5f·$^{1}/_{7}$3-Cu (calc.)** | 65.18 | 6.08 | 6.91 | 9.43 |
| 21 | **5g (found)** | 6.87 | 1.54 | 1.31 | **5.24** |
| 22 | **5g (calc.)** | 51.95 | 6 | 7.57 | 6.86 |
| 23 | **5g·½3-Cu (calc.)** | 49.96 | 7.16 | 9.71 | 5.15 |
| 24 | **11a (found)** | 10.02 | 1.3 | 1.95 | **5.14** |
| 25 | **11a (calc.)** | 66.46 | 5.58 | 11.72 | 5.67 |
| 26 | **11b (found)** | 7.78 | 1.23 | 1.38 | **5.64** |
| 28 | **11b (calc.)** | 69.19 | 5.12 | 9.49 | 7.29 |
| 29 | **11b·3-Cu (calc.)** | 59.80 | 6.83 | 11.16 | 5.36 |
| 30 | **11c (found)** | 4.21 | 0.7 | 0.89 | **4.73** |
| 31 | **11c (calc.)** | 57.22 | 4.24 | 11.77 | 4.86 |
| 32 | **15 (found)** | 11.63 | 1.37 | 1.90 | **6.12** |
| 33 | **15 (calc.)** | 62.69 | 6.88 | 11.25 | 5.57 |
| 34 | **15·3-Cu (calc.)** | 60.36 | 6.69 | 10.06 | 6.00 |
